# Dynamic steps in receptor tyrosine kinase mediated activation of class IA phosphoinositide 3-kinases (PI3K) captured by H/D exchange (HDX-MS)

**DOI:** 10.1016/j.jbior.2012.09.005

**Published:** 2013-01

**Authors:** John E. Burke, Roger L. Williams

**Affiliations:** Medical Research Council, Laboratory of Molecular Biology, Cambridge CB2 0QH, UK

## Abstract

The catalytic subunits of all class IA phosphoinositide 3-kinases (PI3Ks) associate with identical p85-related subunits and phosphorylate PIP2 yielding PIP3, but they can vary greatly in the signaling pathways in which they participate. The binding of the p85 subunit to the p110 catalytic subunits is constitutive, and this inhibits activity, but some of the inhibitory contacts are reversible and subject to regulation. Interaction with phosphotyrosine-containing peptides (RTK-pY) releases a subset of these inhibitory contacts. Hydrogen/deuterium exchange mass spectrometry (HDX-MS) provides a map of the dynamic interactions unique to each of the isotypes. RTK-pY binding exposes the p110 helical domains for all class IA enzymes (due to release of the nSH2 contact) and exposes the C-lobe of the kinase domains of p110β and p110δ (resulting from release of the cSH2 contact). Consistent with this, our in vitro assays show that all class IA isoforms are inhibited by the nSH2, but only p110β and p110δ are inhibited by the cSH2. While a C2/iSH2 inhibitory contact exists in all isoforms, HDX indicates that p110β releases this contact most readily. The unique dynamic relationships of the different p110 isozymes to the p85 subunit may facilitate new strategies for specific inhibitors of the PI3Ks.

## Introduction

The 3-phosphorylated phosphoinositide second messengers have widespread roles in cell signaling. Their involvement in human diseases such as cancer, diabetes, auto-immunity and inflammation has made the phosphatidylinositol 3-kinases (PI3Ks) that produce these lipid second messengers the target of intense efforts at inhibitor development. In mammals, the PI3Ks consist of eight enzymes (Vadas et al., 2011). However, all of these enzymes are closely related and have diverged from an ancestral PI3K known as Vps34, which catalyzes the production of PtdIns(3)P and has been found in all eukaryotes. Although mammalian cells, like other eukaryotes, use PtdIns(3)P in regulating intracellular sorting, they have evolved the four class I PI3Ks, which produce the second messenger PtdIns(3,4,5)P3. While this lipid is only a minor constituent of the plasma membrane, its levels can be quickly modulated by activation of class I PI3Ks and the lipid phosphatase PTEN. The canonical PI3K signaling pathway starts with receptors that activate the PI3Ks to produce PtdIns(3,4,5)P3 and this lipid in turn recruits a range of effector proteins with modules, such as PH domains, that have evolved to recognize this second messenger. The master protein kinase PKB is the best studied and most prominent of these effectors. Phosphorylation by activated PKB of a series of downstream effectors has a key role in cell survival, growth, protein synthesis and the cell cycle.

The class IA PI3Ks consist of p110α, p110β and p110δ catalytic subunits, which associate tightly with a p85-related regulatory subunit. The domain organizations (from N- to C-terminus) of the p110 catalytic subunits are identical: adaptor-binding domain (ABD), Ras-binding domain (RBD), C2 domain, helical domain and kinase domain (Walker et al., 1999; Huang et al., 2007; Berndt et al., 2010; Zhang et al., 2011). The p85 regulatory subunit consists of a BH domain, an SH3 domain, an N-terminal SH2 domain (nSH2), an iSH2 domain and a C-terminal SH2 domain (cSH2) (Breeze et al., 1996; Liang et al., 1996; Musacchio et al., 1996; Nolte et al., 1996; Hoedemaeker et al., 1999; Weber et al., 2000). The ABD binds with high affinity to the iSH2, and this constitutive interaction is essential to stabilize the catalytic subunit in cells. In addition to this high affinity interaction, the p85 also makes weaker interactions with the catalytic subunit that greatly down-regulate the basal activity of the catalytic subunit. The PIK3CA gene encoding the p110α catalytic subunit is one of the most commonly mutated genes in human tumors (Samuels et al., 2004; Chalhoub and Baker, 2009). The gain-of-function mutations in the gene are scattered in all domains of PIK3CA (there are only a few mutations in the RBD, and it is not known whether they are gain-of-function). The two most common mutations are in the helical domain at residue E545 and the C-terminal lobe of the kinase domain at residue H1047. Simultaneous mutation of these two residues has a synergistic effect in activating p110α, suggesting that they act independently (Zhao and Vogt, 2008). The wide distribution of the oncogenic mutations has made it unclear as to the mechanism of up-regulation of PI3K in cancers, however, some of the up-regulating mutations were shown to increase the affinity of the enzyme for lipid membranes (Mandelker et al., 2009; Burke et al., 2012; Hon et al., 2012). Several mutations in the p85 regulatory subunit have also been shown to be oncogenic (Jaiswal et al., 2009; Sun et al., 2010; Urick et al., 2011).

The minimal p85 construct capable of fully down-regulating the basal activity of all three class IA isozymes (p110α, p110β and p110δ) consists of the nSH2, iSH2 and cSH2 domains (Yu et al., 1998; Miled et al., 2007; Burke et al., 2011; Zhang et al., 2011). SH2 domains are well-characterized modules for interacting with phosphotyrosine-containing peptides (Huang et al., 2008), and the SH2 domains of the p85 subunit facilitate interaction of the class IA PI3Ks with receptor tyrosine kinases and adaptor proteins that contain tyrosine-phosphorylated YXXM motifs (Carpenter et al., 1993). These pYXXM motifs interact with the canonical phosphotyrosine peptide-binding site on the SH2 domains, and they compete with p110 for binding to the SH2 domains. It is common that receptors capable of activating PI3Ks have two YXXM motifs, and tandem pYXXM motifs lead to much greater activation than single motifs (Carpenter et al., 1993). Early studies had shown that the nSH2 contacts the helical domain at the site of the E545, one of the oncogenic hotspot mutations for the p110α catalytic subunit (Miled et al., 2007). Subsequently, crystallographic analyses of p110/p85 complexes have shown the basic architecture of the inter-subunit interactions, with the p85 nSH2 contacting the p110 helical domain (Mandelker et al., 2009) and the p85 cSH2 contacting the p110 kinase domain (Zhang et al., 2011). It is clear from these studies that the two SH2 domains interact in distinct ways with p110 subunits, yet both domains are forced to disengage the inhibitory grip on the catalytic subunit in the presence of YXXM-containing peptides. Mutations of residues in the helical domain that are in contact with the nSH2 are activating due to a loss of these inhibitory contacts (Miled et al., 2007). The lack of a structure for any full-length p110/p85 heterodimer has made understanding the structural basis for regulation of p110 by p85 challenging, however, the use of hydrogen deuterium exchange mass spectrometry (HDX-MS) has allowed us to probe p85 regulation of the class IA holoenzymes.

Hydrogen deuterium exchange mass spectrometry (HDX-MS) has become a powerful method to determine dynamic structural perturbations in proteins (Engen, 2009). The technique is used to determine the exchange rate of amide hydrogens with solvent, and this exchange is strongly dependent on the involvement of these amide hydrogens in secondary structure and exposure to solvent. The time scale of changes in amide protection observed by HDX-MS (seconds to hours) is consistent with regions that are likely to be involved in slow conformational fluctuations. Recent results using HDX-MS to examine PI3Ks showed for p110δ how interactions with the RTK phosphopeptides increase PI3K activity by increasing membrane binding (Burke et al., 2011). This study also demonstrated that p110δ, like p110β, interacts with and is inhibited by both the nSH2 and cSH2 domains of p85. The prominent effect of phosphorylated RTK peptide binding is to cause a release of inhibitory contacts and to facilitate interaction with lipid membranes (Burke et al., 2011, 2012; Hon et al., 2012). For p110α, oncogenic mutations throughout the subunit produce changes in exposure that mimic and enhance the dynamic events that occur in the natural activation mechanism of the wild-type enzyme (Burke et al., 2012). Previous crystallographic studies have clearly demonstrated the nature of the constitutive interactions between the p110 subunit and the iSH2 domain of the p85 subunit. In vitro assays have demonstrated that the class IA enzymes differ in their affinity for substrate and their maximal catalytic rates (Beeton et al., 2000). We have now examined the dynamics of the p110/p85 interactions for all three class IA isozymes using HDX-MS and correlated this with enzymatic activity. Our HDX-MS has enabled us to map dynamic footprints of the p85 subunit on each of the p110 subunits and vice versa. This has provided insight into the weaker, regulated interactions between the subunits. These results demonstrate that each of the class IA enzymes has a unique dynamic relationship with the p85 subunit. These unique responses to the p85 regulatory subunit may partially account for the signaling pathways in which the members of the class IA PI3Ks function.

## Materials and methods

### ATPase assays

ATPase assays were carried out using the transcreener ADP^2^ fluorescence polarization assay (Bellbrook laboratories). Reactions were performed in 10 μL in 384 well black plates (Corning 3676). The reactions contained 100 μM ATP, and a dilution series of protein concentrations varying from 1 μM to 1 nM. The protein was serially diluted in a buffer containing 50 mM Hepes pH-7.4, 100 mM NaCl, and 2 mM TCEP. Experiments were carried out in the presence and absence of a pY phosphopeptide (mouse PDGFR residues 735–767, with pY740 and pY751 at a final concentration of 2 μM, referred to afterward as pY). Reactions were started by the addition of ATP (final concentration 100 μM) in a buffer that gave a final concentration of 50 mM Hepes pH-7.4, 100 mM NaCl, 3 mM MgCl_2_, 1 mM EGTA, and 2 mM TCEP. Reactions were allowed to run for 60 min with shaking at 500 rpm. Reactions were stopped using 10 μl of the transcreener stop buffer (1X Stop & Detect Buffer B, 4 nM ADP Alexa633 Tracer, 109 μg/ml ADP2 antibody). This was allowed to equilibrate for 30 min and then the plate was read using a PHERAStar plus HTS microplate reader (BMG Labtech), using a fluorescence polarization module with excitation centered at 633 nm and emission at 650 nm.

### Lipid kinase assays

The lipid kinase activity was determined using a modified membrane capture assay measuring production of 32P-labeled PIP3 (Knight et al., 2007). Lipid vesicles were used at a final concentration of 1 mg/mL and were composed of 5% brain PIP2, 20% brain PS, 45% brain PE, 15% brain PC, 10% cholesterol, and 5% sphingomyelin (Avanti Polar Lipids). The lipid solution and RTK-pY (if relevant) was mixed with each protein construct at a final concentration of enzyme in the reaction of 10 nM, with a final buffer containing 3 mM MgCl2, 1 mM EGTA, 20 mM Tris pH 7.5, 50 mM NaCl, and 50 mM KCl. PI3K phosphopeptide and lipid were allowed to pre-incubate for 10 min at room temperature before the start of the reaction. Reactions were started by adding 100 μM ATP (final concentration) containing 0.1 μCi/μL of [γ32P]-ATP in a total volume of 10 μL. This reaction was carried out for 10 and 30 min, and stopped by mixing 3 μL of the reaction mixture with 3 μL of 20 mM EDTA. Three microliters of this mixture was then spotted on a Hybond-C nitrocellulose membrane (GE healthcare). The membrane was washed six times with a 1 M NaCl/1% phosphoric acid solution. After completion of the last wash, the membrane was dried for 1 h, followed by a 5–20 min exposure to a phosphor screen (Molecular Dynamics). The spot intensity on the phosphor screen was imaged using a Typhoon phosphoimager (GE Healthcare) and quantitated using ImageQuant (GE Healthcare). For experiments determining the phosphopeptide sensitivity of each isoform, a solution containing the indicated amount of pY along with lipid vesicles was added as described above to each isoform.

### HDX measurements

HDX reactions were initiated in HDX buffer (10 mM Hepes pH-7.4, 100 mM NaCl, and 2 mM TCEP) by the addition of proteins (3 μM final) to either pY solution (15 μM pY final) or just pY buffer. This was allowed to preincubate for at least 15 min followed by the addition of 88% D_2_O solution (10 mM Hepes pD-7.4, 50 mM NaCl, and 2 mM TCEP), resulting in a final concentration of 78% D_2_O. “On exchange” reactions were carried out for 3, 30, 300, and 3000 s at 23 °C, followed by addition of a quench buffer, giving a final concentration 0.8% formic acid and 0.5 M Guanidine–HCl. Samples were frozen in liquid nitrogen, and stored at −80 °C until mass analysis.

### Protein expression and purification

Proteins were expressed and purified as described previously (Burke et al., 2011).

### Lipid vesicle preparation

Lipid vesicles were prepared as described previously (Burke et al., 2011).

### Measurement of deuterium incorporation

Samples were rapidly thawed on ice and injected onto a UPLC system immersed in ice as described previously (Burke et al., 2011). The protein was passed over an immobilized pepsin column (Applied Biosystems, Poroszyme^®^, 2-3131-00) at 130 μL/min, and onto a particle van-guard trap (Waters) for 3 min. The trap was then eluted onto an Acquity^®^ 1.7 μm particle, 100 mm × 1 mm C18 UPLC column (Waters) using a 5–45% gradient (buffer A 0.1% formic acid and buffer B 100% acetonitrile) over 20 min and injected into an LTQ Orbitrap XL (Thermo Scientific) acquiring from 350 to 1500 *m*/*z*, with an ESI source operated at a capillary temperature of 225 °C, and a spray voltage of 3.5 kV.

### Protein digestion and peptide identification

**Fig. S1:**
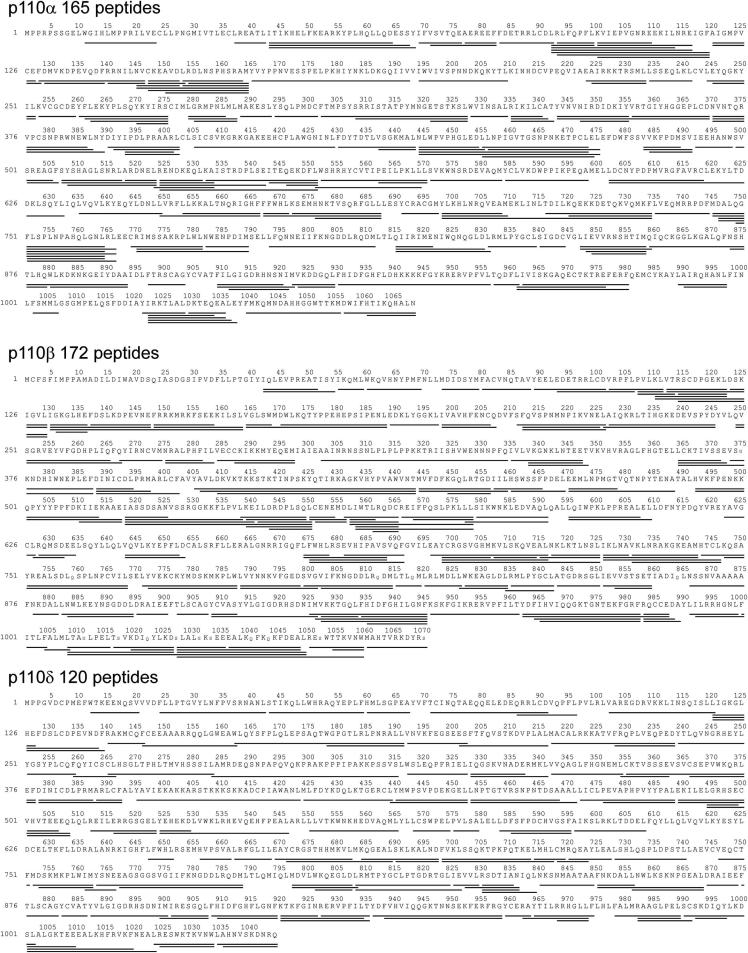
Pepsin digested peptide coverage map of p110α, p110β, and p110δ. Identified and analyzed pepsin-digested peptides are shown under the primary sequence of p110 catalytic isoforms. Peptide digestion protocols was optimized for WT full length p110/p85α heterodimers, and allowed for the identification of 165 peptides for p110α, 172 peptides for p110β, and 120 peptides for p110δ.

**Fig. S2:**
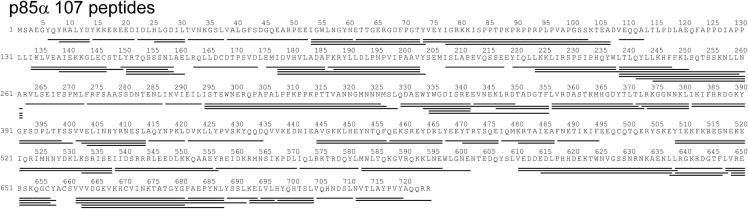
Pepsin digested peptide coverage map of p85α. Identified and analyzed pepsin-digested peptides are shown under the primary sequence of the p85 regulatory subunit. Peptide digestion protocols was optimized for WT full length p110/p85 heterodimers, and allowed for the identification of 107 peptide for p85α.

Peptide identification and mass analysis of the peptide centroids was performed as described previously, using the Mascot search within Proteome Discoverer (Thermo Scientific) and HD-Examiner (Sierra Analytics) (Burke et al., 2011). Mascot identification thresholds were set at 10 ppm for peptide tolerance, and 0.3 Da for fragment MS/MS tolerance. All peptides with a mascot score >20 were analyzed by the HD-Examiner software. All of the peptides were manually validated by searching a non-deuterated protein sample's MS scan to test for correct *m*/*z* state and to check for the presence of overlapping peptides. Ambiguous peptides were excluded from the analysis. The list of all p110 and p85 peptides is shown in Fig. S1 for p110 catalytic subunits and Fig. S2 for p85 regulatory subunits.

### Mass analysis of peptide centroids

Selected peptides were manually examined for deuterium incorporation and accurate identification. Results are presented as relative levels of deuteration with no correction for back exchange, since no fully deuterated protein sample could be obtained. A correction was applied to compensate for differences in the level of deuterium in the exchange buffer (78%). The average error was ≤0.2 Da for corrected data of two replicates. Changes between conditions where the mass difference for a peptide was greater than 6% and greater than 0.5 Da were considered significant. The relative HDX level for every peptide analyzed for every mutant and time point is shown in Fig. S3, and the differences in HDX levels between states are shown in Fig. S5. H/D amide exchange in any peptide reported may be due to any number of amides within the peptide. All samples compared were prepared at the same time, and acquired on the mass spectrometer in the same session.

## Results and discussion

### Basal and pY stimulated activities of p110α, p110β and p110δ

Although all class IA PI3Ks associate with p85-related regulatory subunits, the effect of this association varies among the isotypes. While both p110α and p110β are expressed in most cells, several observations suggest that association with phosphorylated RTKs activates p110α, but has less influence on p110β (Guillermet-Guibert et al., 2008; Kulkarni et al., 2011). One of the unique aspects of the p110β is that it is activated by association with both Gβγ heterodimers and phosphorylated RTKs (Kurosu and Katada, 2001). Using inhibitors selective for p110β and cells derived from a p110β knockout mouse, it has been shown that p110β is responsive to GPCR agonists, while it has only a minor role in signaling downstream of tyrosine kinases (Guillermet-Guibert et al., 2008). It has been proposed that p110β could provide a mechanism for signaling downstream of GPCRs in non-hemopoietic cells where the Gβγ-responsive isotype p110γ is not expressed (Guillermet-Guibert et al., 2008) and that synergy of response by combined GPCR and RTK activation might be the biological role of the p110β isotype (Kurosu and Katada, 2001; Kulkarni et al., 2011).

### Catalysis in the absence of lipid substrates

**Fig. 1:**
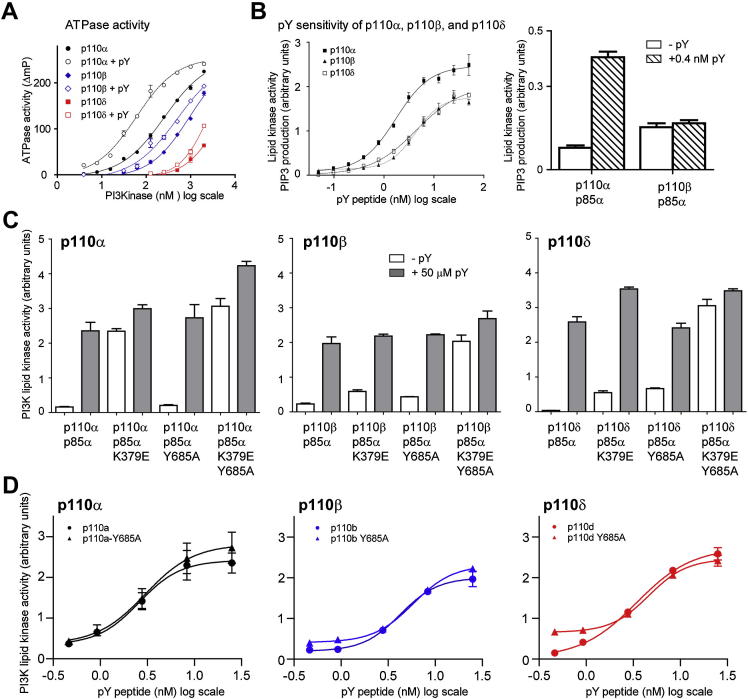
PI3K activity assays. A. ATPase activity of all class IA PI3K isoforms in the absence and presence of bis-phosphorylated PDGFR peptide (pY). This assay is based on displacement of the ADP-Alexa 633 tracer from the ADP^2^ antibody, dependent on the production of ADP. B. Lipid kinase activity assay of all class IA PI3K isoforms with varying amounts of pY (from 0.1 to 100 nM). Assays measured ^32^P-PIP3 production in the presence of 10 nM enzyme, 100 μM ATP, and 5% PIP2 lipid vesicles at a concentration of 1 mg/ml. The amount of lipid kinase activity for p110α and p110β in the presence of 0.4 nM pY is shown in the bar graph. C. The effect of nSH2 (K379E) and cSH2 (Y685A) mutations on lipid kinase activity. The lipid kinase activity of full-length recombinant p110/p85 complexes containing the indicated p85 mutations are shown in the presence and absence of 50 μM pY. D. The pY sensitivity of lipid kinase activity was determined for all class IA PI3K isoforms in the presence of either the wild type or the Y685A mutant of p85 as described above.

**Fig. 2:**
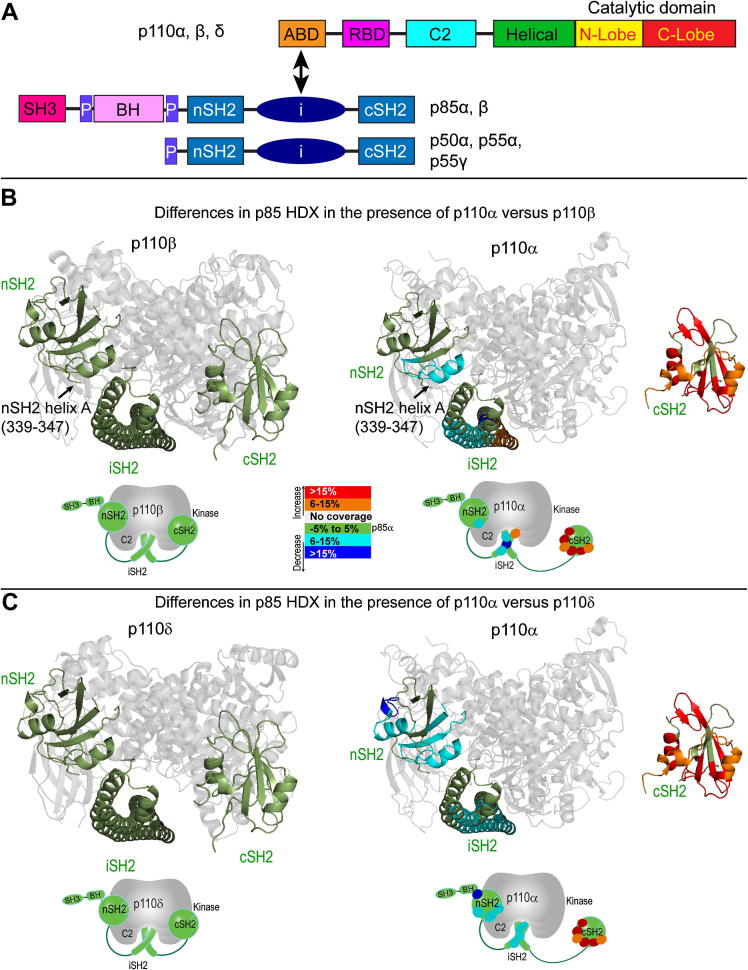
Basal hydrogen/deuterium exchange differences in the p85 regulatory subunit in the presence of different p110 catalytic subunits. A. Domain organization of the different p110 catalytic and p85 regulatory subunits. B. Differences in HDX in p85 between the p110α and p110β complex. In the left panel, a structural model of the nSH2, iSH2, and cSH2 of p85 bound to p110β is shown as a reference (a composite image generated from PDB ID 2Y3A, 3HHM, 1H9O and 2V1Y). In the right panel, peptides spanning p85α that showed both greater than 6% and 0.5 Da changes in H/D exchange in between p85 bound to p110α and p110β are colored on the nSH2, iSH2, and cSH2 and mapped onto a model of p85 bound to p110α (generated from PDB ID 3HHM, 1H9O, and 2V1Y). A simplified schematic representation of HDX differences is shown underneath the structural model. C. Differences in HDX in p85 between the p110α and p110δ complex. In the left panel, structural model of the nSH2, iSH2, and cSH2 of p85 bound to p110δ is shown as a reference (generated from PDB ID 2WXH, 3HHM, 1H9O and 2V1Y). In the right panel, peptides spanning p85α that showed both greater than 6% and 0.5 Da changes in H/D exchange in between p85 bound to p110α and p110δ are colored on the nSH2, iSH2, and cSH2 and mapped onto a model of p85 bound to p110α. (For interpretation of the references to color in this figure legend, the reader is referred to the web version of this article.)

In an effort to understand to what extent the differential signaling roles of the class IA PI3Ks downstream of tyrosine kinases are intrinsic to the enzymes themselves rather than the interactions that they make with signaling components other than phosphorylated tyrosine-containing peptides (pY), we examined the mechanisms of pY-mediated activation of all class IA PI3Ks in vitro. As shown in Fig. 1A, the three class IA isotypes can all act as ATPases, transferring the γ-phosphate from ATP to water in the absence of lipid substrates. Even using this basal activity, it is clear that the three isotypes differ significantly. The p110α/p85α heterodimer has the greatest activity and is about four-fold more active than p110β/p85α. The p110β/p85α is then about three-fold more active than the p110δ/p85α heterodimer. The greater activity of p110α is consistent with the structures of the enzymes. The C-terminal region of all PI3Ks is essential for catalysis. It inhibits the basal activity of the enzyme in the absence of a membrane, and it enhances membrane binding. It has been proposed that this is an important regulatory element of the PI3Ks (Miller et al., 2010). Structures of p110α show a great deal of disorder or irregular structure in this region (Huang et al., 2007; Mandelker et al., 2009; Hon et al., 2012), and this may facilitate entry of water into the active site. In contrast to p110α, the other class IA enzymes have a partially ordered helix in this region that may be more effective in preventing catalysis in the absence of membranes (Zhang et al., 2011). Using an excess of a PDGFR-derived bis-phosphorylated phosphopeptide (pY), the p110α/p85α heterodimer is also activated to a greater extent than the other isotypes. The ability of the pY to activate ATPase activity is likely due to loss of a contact between the 339–347 helix of the nSH2 and helix α10 of the C-lobe of the kinase domain. The HDX-MS results suggest that the 339–347 helix of the nSH2 is more tightly protected in the complex with p110α compared to the complexes with p110β or p110δ (Fig. 2).

### RTK peptide stimulation of PI3K activity in the presence of PIP2-containing membranes

Titration with the pY (PDGFR residues 735–767, with pY740 and pY751) shows that the lipid kinase activity of p110α/p85α complex is stimulated at a lower concentration of the peptide than p110β/p85α or p110δ/p85α heterodimers (Fig. 1B, left panel). Consequently, it is easy to select a condition in which p110α/p85α is activated, while p110β/p85α shows no significant activation (Fig. 1B, right panel). Our in vitro kinase assays suggest that the observation that p110β/p85α does not respond to RTK signaling stimulation in cells may be a result of the inherently lower response of the p110β/p85α heterodimer.

We wanted to test whether this lower p110β/p85α sensitivity to RTK stimulation arises from the influence of the SH2 domains of the p85 subunit. Structural studies have shown that both the nSH2 and cSH2 subunits of p85 interact with p110β and p110δ (Burke et al., 2011; Zhang et al., 2011) and these contacts inhibit the enzymes. In contrast, p110α is fully inhibited by a construct that has only the nSH2 and iSH2 domains (Yu et al., 1998). Although this suggests that the cSH2 has no role in inhibiting p110α, measurements of activity for deletion variants could have complex and unanticipated consequences on the structure of the regulatory subunit and the potential interactions that it could make with the pY outside the SH2 domains. Because of this, we used point mutants in the nSH2 and cSH2 domains that eliminate the contact of these domains with the catalytic subunit (Burke et al., 2011; Zhang et al., 2011). The K379E mutation in the nSH2 eliminates the interaction between the helical domain of the catalytic subunit and the nSH2 by breaking a salt bridge between E545 and K379. In addition to stimulating PI3K in vitro (Miled et al., 2007; Burke et al., 2011; Zhang et al., 2011), this mutation is oncogenic in cells (Sun et al., 2010; Hofmann and Jücker, 2012). The Y685A mutation replaces a tyrosine that is essential for contact of the cSH2 with the C-lobes of the p110β and p110δ kinase domains, thereby eliminating the inhibitory capability of the cSH2, without affecting its ability to bind pY (Burke et al., 2011; Zhang et al., 2011). As shown in Fig. 1C, the K379E mutation increases the basal activity of all three isotypes. In contrast, the Y685A mutation increases the basal activity of only p110β and p110δ. The K379E mutation renders p110α fully active so that no further activation is achieved by addition of pY. Either K379E or Y685A mutants can be further stimulated by pY for both p110β and p110δ. The double mutant K379E/Y685A fully activates all isotypes.

Both HDX-MS and activity assays indicate that the cSH2 makes an inhibitory contact with the catalytic subunit only for p110β and p110δ but not for p110α. The lack of an inhibitory effect of p110α is consistent with a previous report (Yu et al., 1998). However, the same report also showed that bis-phosphorylated IRS-1 activation of p110α/p85 was diminished when the cSH2 was mutated to prevent pY binding. It was proposed that the presence of residues 1–322 (containing the SH3 and BH domains) facilitates a conformational change in p85 such that pY binding to the cSH2 facilitates release of nSH2-mediated inhibition. Our results demonstrating that the Y685A mutation prevents the inhibitory contact of the cSH2 domain with p110β and p110δ, with no influence on p110α, do not exclude such a mechanism, because the Y685A mutation does not affect phosphopeptide binding to the cSH2 (Zhang et al., 2011).

To test whether the lack of the cSH2 inhibitory contact was responsible for the higher pY sensitivity of p110α (Fig. 1B), we examined the pY sensitivity for all three class IA p110 isoforms in the presence of either the wild type or Y685A p85 constructs. We found that the presence of the Y685A mutation had no effect on the pY sensitivity of any isoforms (Fig. 1D). Therefore the mechanism of enhanced sensitivity of p110α relative to the other isoforms remains unknown.

### H/D exchange mass spectrometry of the basal states of class IA enzymes in complexes with p85α

**Fig. S4:**
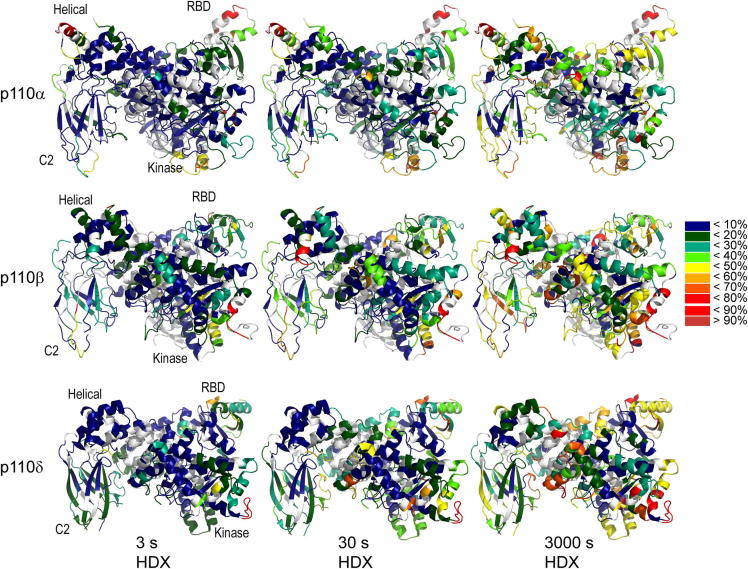
Global H/D levels mapped onto each p110 catalytic isoform. The relative percent deuteration of all peptic peptides shown in Fig. S3 are used to color the structures of the three catalytic isoforms at three timepoints (3HHM, 2Y3A, 2WXH) according to the legend.

**Fig. S5:**
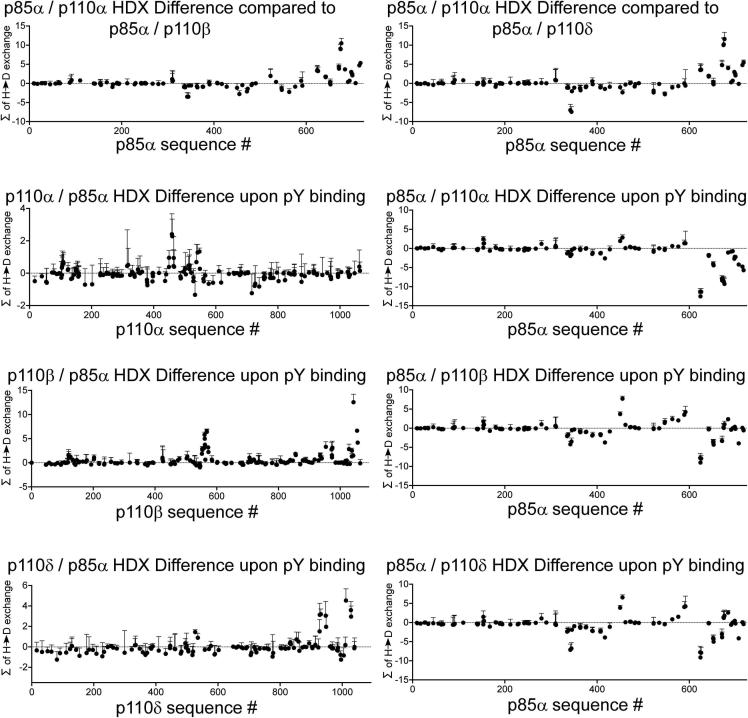
For the entire set of peptides for p85 (107 peptides), p110α (165 peptides), p110β (172 peptides), and p110δ (120 peptides) the integrated # of deuterium difference in exchange at all four timepoints between each condition labeled was measured. Data represent mean ± SD of two independent experiments. The *x* axis was generated by taking the central residue of each peptide.

We have used HDX-MS to investigate the dynamic structural consequences of the differences in interactions between the p85 regulatory subunit and the three class IA enzymes in the basally inhibited state (Fig. 2). We were able to identify a number of peptic peptides for each of the class IA catalytic isoforms (Fig. S1) and the p85 regulatory subunit (Fig. S2). For all peptides, we quantified the relative deuteration level at four timepoints (3, 30, 300, and 3000 s) and the full H/D exchange data is shown in Fig. S3. Experiments were carried both in the presence and absence of pY. The global deuteration level of p110α, p110β and p110δ at three timepoints are shown in Fig. S4. We were able to determine the difference in HDX levels for all three p110/p85 heterodimers in the presence of pY, as well as the difference in the p85 subunits in the presence of different catalytic isoforms (Fig. S5).

#### Comparison of H/D exchange in the cSH2 among class IA heterodimers

Comparing the HDX levels of the p85 subunit in the presence of various catalytic subunits enabled us to uncover differences in how the p110 subunits interact with p85. We find that the main difference between the isoforms is that the cSH2 has much higher H/D exchange rates (i.e., is much more exposed) in the presence of the p110α catalytic subunit compared to p110β and p110δ (Fig. 2A,B). This is consistent with p110α forming no contact with the cSH2 and is in agreement with activity measurements showing no inhibitory influence of the cSH2.

#### Comparison of H/D exchange in the nSH2 among class IA heterodimers

In contrast to the cSH2, interaction with p110α results in decreased HDX in the nSH2 and iSH2 compared with either p110β or p110δ (Fig. 2). The one area in the nSH2 that shows decreased HDX in p110α compared to p110β and p110δ is the helix A (residues 339–347), which is in contact with the C-terminal lobe of the kinase domain in the crystal structure of p110α bound to the niSH2 fragment of p85 (Mandelker et al., 2009; Vadas et al., 2011). This may explain why the nSH2 more strongly inhibits the p110α subunit compared to p110β, and p110δ (Fig. 1C). There is also a large increase in HDX in the C-terminus of the nSH2 (414–420) in p110δ compared to both p110α and p110β. Although there is no crystal structure of the p110β or p110δ interacting with the nSH2, the HDX-MS suggests that there are conformational differences with respect to p110α that result in more protection of the nSH2, likely due to it making more extensive contacts in the p110α/p85 complex.

### H/D exchange mass spectrometry of the pY activated states of the class IA PI3Ks

#### H/D exchange comparison of the helical domains of class IA complexes

**Fig. 3:**
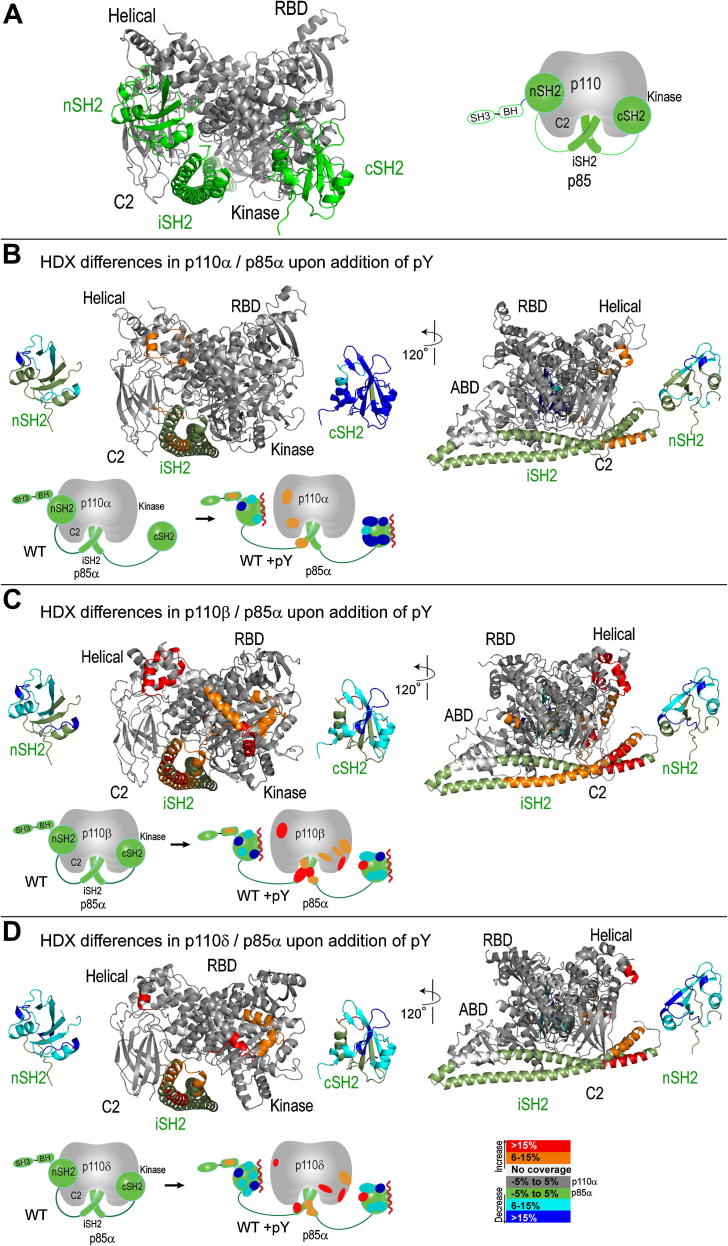
HDX differences in the p110 catalytic and p85 regulatory subunits in the presence of pY. A. The structural model of SH2 binding to the p110 catalytic subunit based on the crystal structures of p110α bound to the niSH2 fragment of p85, and p110β bound to the icSH2 fragment of p85. The p85 is colored green and the p110 subunit is gray. B. Peptides that showed HDX differences in p110α/p85α in the presence of 15 μM pY are mapped and colored onto a model of the p110α/p85α complex. A simplified schematic representation to the right of the structural model helps orient the location of HDX differences. C. Peptides that showed HDX differences in p110β/p85α in the presence of 15 μM pY are mapped and colored onto a model of the p110β/p85α complex. D. Peptides that showed HDX differences in p110δ/p85α in the presence of 15 μM pY are mapped and colored onto a model of the p110δ/p85α complex. (For interpretation of the references to color in this figure legend, the reader is referred to the web version of this article.)

All three class IA PI3Ks show an increase in exchange in the helical domain of the p110 subunit in the presence of 15 μM bis-phosphorylated PDGFR-derived peptide (Fig. 3). This exposure is consistent with the pY causing release of the inhibitory contact of the nSH2 with the helical domain. In addition, pY binding exposes a region near the N-terminal end of the first long helix in the coiled-coil iSH2 domain. This exposure is likely the result of an increase in dynamics of the nSH2/iSH2 linker upon freeing the nSH2 domain from contact with the catalytic subunit. For p110β and p110δ, there is also an increase in exposure of the C-terminal end of the second helix of the iSH2, and this is likely due to freeing the cSH2. No increase in exposure of this helix for p110α is consistent with the cSH2 being already free in the basal state for the p110α/p85 complex.

#### H/D exchange comparison of the C2/iSH2 interfaces of class IA complexes

There is an inhibitory interaction between the C2 domain and the second helix of the iSH2, and mutants that disrupt this interaction, such as mutants of D560 and N564 in the iSH2 domain are oncogenic for all class IA PI3Ks (Zhao and Vogt, 2008; Jaiswal et al., 2009). Mutations of residues at this interface for p110α/p85 result in an increase in HDX in the second helix of the iSH2 as well as for the loop in the C2 domain that contains N345 (Burke et al., 2012). The p110β/p85 complex is unique among the class IA enzymes in that pY binding causes a larger increase in HDX across most of the iSH2 compared to other isozymes (Fig. 3B). Interestingly, we have found that many of the HDX changes we see in the iSH2 for p110β upon pY binding are seen upon membrane binding by the pY-activated p110α, and p110δ (Burke et al., 2011, 2012). These results suggest that all three class IA enzymes undergo similar conformational changes upon full activation, but that these conformational changes are elicited by different stimuli. This implies that the p110β/p85 inhibitory contacts at the C2/iSH2 interface are weaker in the basal state and are consequently disrupted by simple pY binding, whereas p110α, and p110δ require in addition subsequent membrane binding to achieve the same state. This view of the natural activation process gives some insight into the mechanism of up-regulation of PI3Ks in tumors. Oncogenic mutations in p110α mimic the dynamic changes induced by pY binding and by membrane binding in the WT enzyme (Burke et al., 2012).

**Fig. 4:**
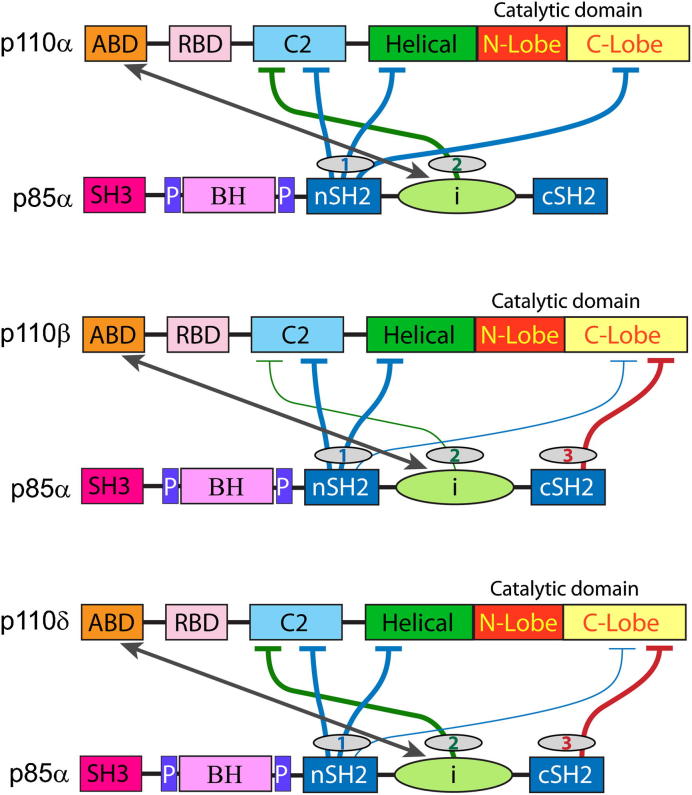
Differential regulation of the class IA PI3Ks by p85. Schematic representation of inhibitory interactions between the p110 and p85 subunits. The p110α subunit has only two inhibitory ‘brakes’ on kinase activity, one from the nSH2 and one from the iSH2. In p110β there are three brakes on lipid kinase activity, due to inhibitory contacts with the nSH2, iSH2, and cSH2, however our HDX results show that the nSH2 and iSH2 ‘brakes’ are slightly disrupted in the basal state compared to p110α. In p110δ there are also three ‘brakes’ on activity from the nSH2, iSH2, and cSH2, however in this case only the nSH2 ‘brake’ is partially disrupted in the basal state, and the iSH2 ‘brake’ remains fully inhibitory.

It has been suggested that p110β represents an activated PI3K relative to p110α based on the observation that p110α undergoes numerous spontaneous gain-of-function mutations in human cancers (Brown and Auger, 2011; Vogt, 2011), and transfection of the wild-type p110α is not oncogenic, whereas transfection of wild-type p110β and p110δ is (Zhao et al., 2005; Kang et al., 2006; Huang et al., 2008). In p110α, residue N345 interacts with D560 and N564, and mutation of residues involved in this interaction leads to increased PI3K signaling and oncogenesis (Huang et al., 2008; Jaiswal et al., 2009; Zhang et al., 2011). It was proposed that this inhibitory cSH2/iSH2 interaction is disrupted in the wild-type p110β relative to p110α due to a sequence difference in p110β relative to p110α at human p110β residue K342 (Dbouk et al., 2010). However, other studies showed that the p85 D564N mutation is up-regulating and oncogenic even for p110β (Jaiswal et al., 2009; Zhang et al., 2011). The structure of the mouse p110β/p85β complex shows that D560 and N564 are nearest to S467 and S468 in p110β and that the residue that was proposed before the structure was reported to be equivalent to K342 in human p110β is actually not at the p110β/p85β interface (Zhang et al., 2011). Our HDX data for p110β indicate that the region of the iSH2 in contact with the C2 is more exposed in p110β compared to either p110α or p110δ (Fig. 2B), and that the addition of pY is sufficient to further expose this region, which does not occur for p110α or p110δ. This indicates that the C2/iSH2 contacts exist in p110β, but they are much more dynamic for p110β, which is consistent with the proposal that p110β is less inhibited by the iSH2 than p110α (Dbouk et al., 2010) (Fig. 4).

#### H/D exchange comparison of the kinase domains among the class IA isozymes

Only p110β and p110δ show an exposure in the C-lobe of the kinase domain in response to pY binding (Fig. 2). Concomitant with exposure of the kinase domain C-lobe is exposure of a peptide in the cSH2 that corresponds to the site that contacts the C-lobe in the crystal structure of the p110β/p85β complex (Zhang et al., 2011). This is the peptide containing Y685 whose mutation leads to p110β and p110δ activation (Fig. 1C). In contrast, the p110α subunit shows no significant changes in exposure of the kinase domain and no exposure of the cSH2 in response to pY binding. These HDX-MS results are consistent with the activity assays for the mutant p85 subunits that show that the cSH2 domain does not inhibit p110α (Fig. 1C). There is no exposure of the nSH2 for any of the class IA complexes, and this is likely because the same region that would become exposed by releasing from the p110 subunit becomes buried in the interaction with the pY. Recent work on p110α containing the E545K mutation, which mimics the release of the nSH2 caused by pY binding showed exposure of the nSH2 confirming this hypothesis (Burke et al., 2012). This contrasts with the cSH2 domain because the site of contact with the p110 subunit and the pY binding sites do not coincide (They are on opposite sides of the cSH2 domain). Only peptides long enough to reach from the pY binding site to the surface of the p110 subunit are capable of displacing the cSH2 domain and thereby activating p110β/p85 (Zhang et al., 2011).

#### H/D exchange in the BH domain

For the three class IA PI3Ks, there is an increase in exposure of the BH domain on pY binding (Fig. 3), suggesting that there may be a contact between the BH domain and another region in the p85 subunit or the p110 subunit that is released on pY binding. The peptide in the BH domain that becomes exposed (residues 149–158) corresponds to a region that is close to the crystallographic dimer interface in the BH domain structure (Musacchio et al., 1996), although the BH domain is not a dimer when expressed in isolation. It may be that the crystal dimer mimics a contact that the domain makes in the context of the full PI3K.

### Interpretation of HDX-MS results in terms of structural dynamics

Although the HDX-MS results can be readily used to estimate the amplitudes of structural fluctuation upon pY binding, there is no information about the direction of the movement. Normal mode analysis of protein structures can be used to computationally estimate conformational fluctuation. The elastic network model of normal mode analysis (Tama and Sanejouand, 2001) uses an extremely simplified potential energy function so that it is computationally tractable. The analysis generates an estimation of the direction of conformational fluctuation that in many cases agrees with experimentally observed changes in structure (Tama and Sanejouand, 2001).

The following are the Supplementary data related to this article:

Movie 1. An illustration of the fluctuation dictated by the lowest frequency normal mode for the p110α/iSH2 and p110β/iSH2 complexes (Tama and Sanejouand, 2001; Suhre and Sanejouand, 2004). The structures used in the analysis are based on PDB ids 3HHM (p110α) and 2Y3A (p110β). The nSH2 and cSH2 domains have been omitted in order to model only what is common to the two complexes. The structures are colored by difference in exchange according to the legend in Fig. 2. The left panel illustrates the p110α/p85α complex and the differences in exchange between the basal state in the absence of membranes and the pY-activated state in the presence of membranes (Burke et al., in press). The right panel illustrates the p110β/p85 the differences in exchange between the basal state and the pY-bound state in the absence of membranes.

In Movie 1, we illustrate the lowest frequency normal modes for p110α and for p110β. For the purpose of these calculations, we have used only the p110/iSH2 complex, since the iSH2 domain is the only domain of p85 that is in common between the p110α and p110β crystal structures. The extremely simplified potential function used for these calculations means that the normal mode is dominated by the shape of the complex (Tama and Sanejouand, 2001). This analysis suggests a fluctuation of the ABD/iSH2 relative to the rest of the p110 subunit. We have previously observed an exposure of the ABD-RBD linker in p110α upon membrane binding that we proposed was a consequence of the ABD moving relative to the rest of the catalytic subunit (Burke et al., 2012). This is consistent with the exposure in the backbone amides of the iSH2 domains that occurs in p110α and p110δ upon membrane binding and in p110β upon pY binding as measured by HDX-MS. In the p110α/p85 heterodimer, there are many mutations of the p110 and p85 subunits that are associated with cancers, and these up-regulating mutations facilitate the closed-to-open transition that makes these mutant enzymes have a greater affinity for lipid membranes (Burke et al., 2011, 2012; Hon et al., 2012). As illustrated in Movie 1, many of the regions where exposure of the p110/p85 complex elicited either by pY and membrane binding for p110α or by pY binding alone for p110β correspond to regions containing oncogenic mutations in p110α. Equivalent oncogenic mutations of p110β are not observed in human cancers, and our HDX-MS results show that p110β in many ways mimics the dynamics that we see in oncogenic mutations of p110α (Burke et al., 2012). This correlates well to the proposal that p110β has naturally acquired the oncogenic mutants seen in p110α (Brown and Auger, 2011). The normal mode analysis suggests that the large-scale conformational motions most easily accommodated by the shape of the complex broadly agree with the conformational changes elicited by the perturbing influences of pY binding, membrane binding and oncogenic mutations.

## Conclusions

The observation that p110α is involved in signaling downstream of receptor tyrosine kinases in preference to p110β may reflect the enhanced sensitivity to pY activation that we see in vitro. This would also be consistent with a model proposed for prostate epithelial cells in which p110α activity is stimulated by insulin whereas p110β provides a baseline level of PIP3 production (Knight et al., 2006; Jia et al., 2008). The role of p110β may be particularly important in a cellular context where it is synergistically activated by Gβγ heterodimers downstream of G-protein coupled receptors. Structural and biochemical methods, including X-ray crystallography and HDX-MS, suggest that class IA PI3K lipid kinase activity is differentially regulated by ‘brakes’ provided by the nSH2, iSH2, and cSH2 of the p85 regulatory subunit (Fig. 4). Our results also suggest that there are stereotypical conformational changes within the class I PI3Ks that accompany activation by RTK pY peptides and membrane binding and that these conformational changes can be induced by oncogenic mutations. Because these activating influences converge on membrane interaction, the HDX-MS results could act as a roadmap to design inhibitors that would work by preventing the closed-to-open transition that occurs upon activation and membrane binding. Given the wealth of structural information available for the class I PI3Ks, approaches such as tethering (Hyde et al., 2003; Raimundo et al., 2004; Sadowsky et al., 2011) could be taken to develop small molecule inhibitors that would lock the enzyme in the closed state.

## Appendix A Supplementary material

Fig. S3 All HDX data for all peptides analyzed for p110α, p110β, p110δ and p85α in the presence and absence of pY. The relative percent deuteration for each complex at each time point is shown and colored according to the legend. The standard deviations of each time point are shown in the columns to the right. The charge state (*Z*), residue start number, residue end number, as well as the retention time (RT) are displayed for every peptide. The four timepoints (3, 30, 300, and 3000 s) are labeled for the conditions tested. The data listed are the average of two independent experiments performed on the same day.
