# Supplementary figures and images for: Dynamic steps in receptor tyrosine kinase mediated activation of class IA phosphoinositide 3-kinases (PI3K) captured by H/D exchange (HDX-MS)
Source: Adv Biol Regul. 2013 Jan;53(1):97–110. doi: 10.1016/j.jbior.2012.09.005 (PMC3613897; doi:10.1016/j.jbior.2012.09.005)

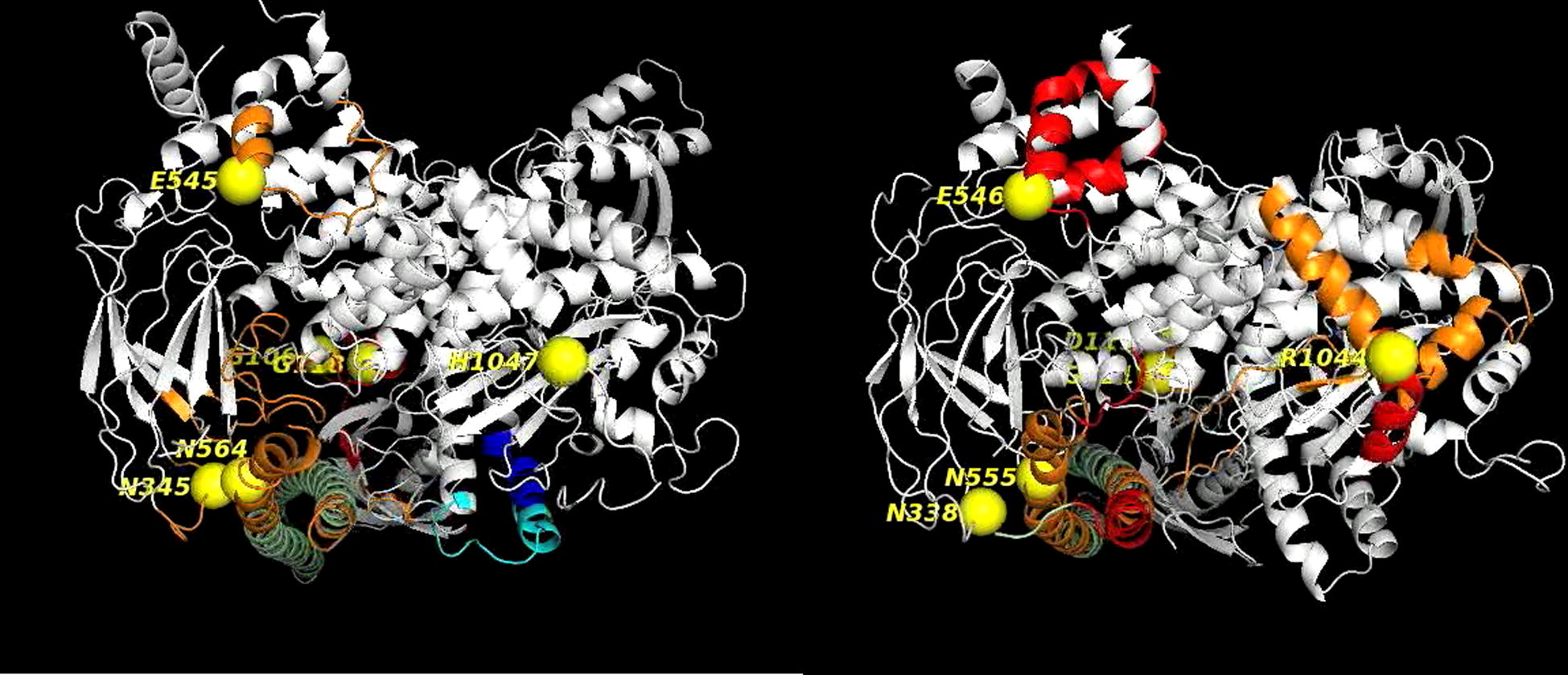

Supplement: Supplementary file 1 — Movie 1. An illustration of the fluctuation dictated by the lowest frequency normal mode for the p110α/iSH2 and p110β/iSH2 complexes (Tama and Sanejouand, 2001; Suhre and Sanejouand, 2004). The structures used in the analysis are based on PDB ids 3HHM (p110α) and 2Y3A (p110β). The nSH2 and cSH2 domains have been omitted in order to model only what is common to the two complexes. The structures are colored by difference in exchange according to the legend in Fig. 2. The left panel illustrates the p110α/p85α complex and the differences in exchange between the basal state in the absence of membranes and the pY-activated state in the presence of membranes (Burke et al., in press). The right panel illustrates the p110β/p85 the differences in exchange between the basal state and the pY-bound state in the absence of membranes. [file mmc5.jpg]
