# Supplementary material for: Dynamic steps in receptor tyrosine kinase mediated activation of class IA phosphoinositide 3-kinases (PI3K) captured by H/D exchange (HDX-MS)
Source: Adv Biol Regul. 2013 Jan;53(1):97–110. doi: 10.1016/j.jbior.2012.09.005 (PMC3613897; doi:10.1016/j.jbior.2012.09.005)
Supplement: Fig. S3 — All HDX data for all peptides analyzed for p110α, p110β, p110δ and p85α in the presence and absence of pY. The relative percent deuteration for each complex at each time point is shown and colored according to the legend. The standard deviations of each time point are shown in the columns to the right. The charge state (Z), residue start number, residue end number, as well as the retention time (RT) are displayed for every peptide. The four timepoints (3, 30, 300, and 3000 s) are labeled for the conditions tested. The data listed are the average of two independent experiments performed on the same day. [file mmc1.pdf]

					GLOBAL HDX LEVELS p110 alpha / p85 alpha				p110 alpha peptides p110 alpha / p85 alpha + pY				STDEVS p110 alpha / p85 alpha				p110 alpha / p85 alpha + pY			
Start	End	CS	#D	RT	3	30	300	3000	3	30	300	3000	3	30	300	3000	3	30	300	3000
11	23	3	9	14.23-15.10	12%	26%	37%	44%	10%	26%	36%	42%	0%	1%	1%	1%	0%	0%	1%	0%
37	42	2	4	8.01-8.32	0%	-1%	1%	15%	0%	-1%	0%	12%	0%	0%	0%	1%	0%	0%	0%	2%
43	64	3	19	10.82-11.04	6%	15%	27%	38%	5%	15%	26%	36%	0%	1%	0%	0%	0%	0%	0%	1%
43	67	3	22	10.95-11.17	4%	13%	25%	35%	4%	13%	24%	34%	0%	1%	0%	1%	0%	0%	1%	1%
43	68	3	23	11.56-11.86	4%	13%	24%	33%	4%	13%	23%	32%	0%	1%	0%	2%	0%	0%	0%	1%
69	76	1	6	10.65-10.91	0%	2%	13%	16%	0%	3%	13%	16%	0%	0%	0%	1%	0%	1%	0%	0%
71	82	2	10	10.61-10.82	2%	13%	24%	27%	2%	14%	24%	26%	0%	1%	1%	1%	0%	0%	0%	1%
77	82	1	4	8.32-8.49	2%	16%	24%	24%	2%	17%	24%	23%	0%	2%	1%	1%	0%	0%	0%	1%
83	92	2	8	11.47-11.69	1%	1%	3%	8%	0%	1%	2%	8%	0%	0%	0%	0%	0%	0%	0%	0%
92	99	1	5	16.39-16.65	0%	0%	0%	2%	0%	0%	0%	2%	0%	0%	0%	0%	0%	0%	0%	0%
92	113	3	18	14.06-14.49	10%	17%	25%	31%	10%	17%	25%	30%	0%	1%	0%	1%	0%	1%	1%	1%
92	116	5	21	13.20-13.84	11%	18%	29%	39%	11%	19%	30%	38%	0%	0%	1%	1%	0%	0%	0%	1%
92	119	4	24	14.80-15.27	10%	16%	26%	36%	10%	16%	27%	36%	0%	0%	0%	1%	0%	0%	0%	1%
93	99	1	4	14.88-15.40	1%	0%	1%	1%	0%	1%	1%	1%	0%	0%	0%	0%	0%	0%	0%	0%
93	119	4	23	13.80-14.32	10%	16%	26%	37%	9%	17%	28%	37%	0%	0%	1%	0%	0%	0%	0%	1%
100	109	2	7	5.56-5.82	33%	49%	58%	57%	32%	51%	57%	56%	1%	1%	2%	3%	1%	1%	1%	1%
100	116	4	14	7.84-8.06	13%	21%	38%	54%	13%	24%	41%	55%	0%	0%	1%	1%	0%	0%	1%	0%
100	119	2	17	11.17-11.47	9%	16%	29%	43%	8%	17%	32%	43%	0%	1%	0%	1%	0%	0%	0%	1%
120	127	1	5	11.17-11.47	5%	15%	30%	45%	5%	17%	31%	46%	0%	1%	0%	1%	0%	0%	0%	1%
120	128	1	6	15.23-15.44	3%	14%	32%	45%	3%	15%	33%	47%	0%	1%	1%	2%	0%	0%	1%	2%
128	146	4	16	13.37-13.71	7%	16%	25%	28%	7%	16%	25%	27%	0%	1%	0%	1%	0%	0%	0%	1%
129	139	2	8	10.00-10.26	11%	20%	36%	39%	10%	21%	35%	36%	0%	1%	1%	1%	0%	0%	0%	1%
140	146	2	5	9.31-9.44	8%	24%	35%	37%	6%	23%	36%	36%	1%	2%	2%	0%	0%	0%	1%	3%
145	152	1	6	5.78-5.87	1%	1%	3%	30%	1%	1%	3%	34%	0%	0%	0%	1%	0%	0%	0%	0%
145	164	3	17	9.44-9.57	9%	11%	19%	41%	8%	11%	18%	42%	0%	1%	0%	2%	1%	0%	0%	0%
147	152	1	4	3.91-4.00	0%	0%	3%	34%	0%	1%	3%	39%	0%	0%	0%	0%	0%	0%	0%	1%
147	153	1	5	8.88-8.97	1%	1%	5%	37%	1%	2%	4%	40%	0%	1%	0%	2%	0%	0%	0%	5%
147	164	3	15	8.84-9.01	10%	12%	22%	42%	9%	12%	21%	43%	0%	0%	1%	1%	0%	0%	1%	1%
150	164	3	12	9.14-9.35	12%	15%	28%	47%	11%	16%	27%	47%	0%	0%	1%	1%	0%	0%	0%	1%
153	163	3	8	4.65-4.88	16%	21%	34%	41%	16%	23%	32%	39%	0%	1%	2%	1%	1%	1%	1%	1%
153	164	3	9	6.09-6.30	12%	16%	27%	38%	12%	17%	26%	38%	0%	0%	1%	1%	0%	1%	1%	1%
154	164	2	8	4.74-4.97	14%	18%	29%	41%	13%	20%	28%	39%	1%	0%	2%	1%	0%	0%	2%	1%
165	192	3	22	11.51-11.82	19%	28%	37%	46%	18%	28%	35%	45%	0%	0%	1%	1%	0%	0%	1%	1%
194	209	3	13	12.98-13.11	16%	31%	40%	42%	15%	30%	39%	39%	1%	1%	1%	3%	1%	0%	1%	1%
223	233	2	9	5.42-5.65	36%	42%	45%	47%	35%	42%	44%	48%	0%	1%	0%	1%	1%	2%	0%	2%
224	233	3	8	4.56-4.97	33%	39%	42%	43%	34%	40%	41%	42%	1%	1%	2%	2%	1%	3%	1%	2%
234	241	2	6	10.65-10.95	71%	72%	72%	72%	69%	72%	72%	72%	1%	1%	1%	1%	2%	1%	1%	1%
244	252	2	7	12.33-12.55	43%	53%	53%	53%	41%	55%	53%	53%	0%	1%	1%	2%	1%	1%	1%	2%
245	252	2	6	11.60-11.77	29%	41%	42%	42%	28%	43%	42%	41%	0%	0%	1%	1%	0%	1%	1%	1%
253	259	1	5	5.51-5.65	2%	6%	10%	12%	1%	6%	10%	12%	0%	0%	1%	0%	0%	1%	0%	1%
260	275	4	13	11.86-12.38	12%	14%	16%	25%	12%	15%	16%	24%	0%	0%	0%	0%	0%	0%	0%	0%
261	275	4	12	10.87-11.26	12%	14%	16%	21%	12%	14%	15%	20%	0%	0%	0%	0%	0%	0%	0%	0%
262	275	2	11	9.96-10.18	10%	11%	12%	15%	10%	11%	11%	14%	0%	1%	0%	1%	0%	0%	0%	0%
270	275	2	4	6.43-6.65	18%	20%	21%	26%	18%	20%	20%	25%	0%	1%	1%	0%	1%	0%	0%	2%
279	285	2	4	10.87-11.04	22%	26%	44%	46%	22%	27%	40%	45%	0%	1%	5%	3%	0%	0%	1%	3%
279	287	2	6	14.02-14.06	13%	17%	23%	27%	14%	17%	23%	25%	0%	1%	0%	2%	0%	0%	0%	1%
280	287	1	5	13.02-13.37	14%	17%	26%	26%	13%	18%	26%	25%	0%	1%	1%	1%	0%	0%	0%	1%
288	293	1	4	6.88-7.18	33%	37%	52%	67%	31%	38%	50%	65%	0%	1%	1%	1%	0%	1%	1%	1%
294	301	1	5	11.77-11.99	37%	51%	64%	63%	36%	52%	63%	62%	0%	1%	1%	2%	0%	1%	1%	1%
302	327	3	22	11.08-11.26	41%	44%	45%	47%	40%	46%	45%	46%	1%	3%	1%	2%	1%	1%	1%	1%
308	327	3	17	8.62-8.92	48%	50%	54%	57%	47%	53%	54%	58%	0%	1%	1%	1%	1%	0%	1%	1%
321	327	1	5	5.42-5.65	23%	40%	51%	67%	22%	41%	52%	65%	0%	3%	2%	1%	0%	0%	0%	1%
328	334	1	5	14.15-14.41	31%	38%	40%	42%	31%	39%	41%	44%	1%	0%	1%	1%	0%	1%	1%	2%
335	340	2	4	9.48-9.66	0%	0%	-1%	3%	-1%	0%	-1%	2%	0%	1%	1%	1%	0%	0%	0%	1%
335	341	2	5	9.57-9.79	0%	0%	1%	13%	0%	0%	1%	11%	0%	0%	0%	0%	0%	0%	0%	0%
335	342	1	6	9.10-9.31	0%	2%	14%	26%	0%	2%	12%	23%	0%	0%	0%	1%	0%	0%	0%	0%
343	355	2	11	12.55-12.64	34%	50%	55%	56%	35%	52%	55%	56%	0%	1%	1%	2%	0%	0%	0%	1%
343	369	3	24	12.81-12.98	15%	22%	24%	25%	15%	23%	24%	24%	0%	0%	1%	1%	0%	1%	1%	1%
347	355	2	7	10.22-10.48	26%	37%	44%	47%	23%	38%	46%	47%	1%	0%	0%	1%	0%	0%	1%	1%
356	369	2	11	9.23-9.31	2%	5%	6%	7%	3%	5%	6%	6%	0%	0%	0%	1%	0%	0%	0%	0%
370	386	2	13	12.12-12.33	10%	26%	38%	46%	9%	27%	36%	45%	0%	2%	1%	1%	0%	1%	0%	1%
370	387	3	14	13.71-13.97	8%	23%	34%	40%	7%	23%	32%	39%	0%	1%	1%	2%	0%	1%	1%	1%
370	389	3	16	13.76-13.97	7%	21%	32%	40%	6%	20%	31%	38%	0%	2%	1%	1%	0%	0%	1%	1%
390	402	2	9	13.02-13.41	1%	3%	10%	11%	1%	1%	7%	11%	0%	0%	0%	0%	0%	0%	0%	0%
391	402	3	8	12.55-12.81	1%	1%	1%	2%	1%	1%	1%	2%	0%	0%	0%	0%	0%	0%	0%	0%
393	401	2	5	12.55-12.76	0%	0%	0%	1%	1%	0%	0%	1%	0%	0%	0%	0%	1%	0%	0%	0%
393	402	2	6	13.24-13.45	0%	1%	5%	6%	0%	0%	3%	5%	1%	0%	0%	0%	0%	0%	0%	1%
405	429	3	22	11.95-12.29	3%	3%	5%	8%	3%	4%	5%	8%	0%	0%	0%	1%	0%	0%	0%	0%
430	443	2	12	13.84-14.15	8%	17%	25%	32%	7%	17%	24%	30%	0%	1%	1%	1%	0%	0%	0%	1%
437	443	1	5	8.79-8.97	18%	34%	39%	39%	16%	35%	39%	39%	1%	1%	2%	1%	0%	0%	0%	1%
444	454	1	7	13.54-13.67	13%	24%	37%	37%	14%	31%	37%	35%	0%	2%	1%	2%	0%	1%	1%	1%
444	455	1	8	15.36-15.53	12%	27%	44%	43%	15%	37%	43%	42%	1%	1%	1%	1%	0%	0%	0%	1%
444	473	2	23	16.48-16.87	8%	18%	33%	42%	9%	23%	34%	45%	0%	1%	1%	1%	0%	0%	1%	1%
444	475	3	25	17.57-17.92	6%	15%	28%	35%	7%	19%	29%	38%	0%	1%	0%	1%	0%	0%	1%	1%
455	473	2	14	12.38-12.64	9%	17%	34%	51%	9%	18%	35%	52%	0%	1%	1%	2%	0%	0%	1%	1%
455	474	2	15	11.73-11.99	8%	15%	32%	48%	8%	17%	33%	52%	0%	1%	1%	3%	0%	0%	1%	1%
456	475	2	15	13.07-13.54	8%	15%	29%	44%	7%	16%	30%	45%	0%	1%	1%	2%	0%	0%	0%	1%
483	489	2	4	11.90-12.03	17%	18%	22%	26%	15%	18%	21%	23%	0%	1%	0%	2%	0%	0%	2%	2%
483	491	2	6	12.20-12.85	24%	28%	43%	46%	24%	28%	40%	44%	0%	1%	0%	1%	0%	1%	1%	1%
484	491	2	5	11.99-12.29	29%	32%	50%	53%	28%	32%	47%	51%	1%	1%	1%	2%	0%	0%	1%	1%
492	498	1	5	9.66-9.83	13%	35%	38%	38%	12%	37%	39%	38%	0%	0%	1%	2%	0%	1%	1%	1%
499	505	1	5	3.53-3.67	76%	73%	75%	73%	76%	77%	74%	75%	1%	4%	3%	1%	1%	6%	2%	2%
501	506	1	4	8.49-8.58	78%	77%	77%	79%	78%	78%	76%	76%	3%	3%	2%	2%	1%	0%	2%	1%
507	517	3	9	8.45-8.66	40%	38%	39%	39%	40%	41%	41%	40%	0%	1%	1%	1%	0%	1%	0%	2%
507	523	2	15	8.88-9.18	41%	41%	42%	41%	41%	43%	42%	41%	1%	1%	1%	1%	0%	0%	0%	1%
508	523	2	14	8.79-9.01	45%	43%	45%	44%	45%	47%	45%	45%	1%	1%	0%	2%	1%	0%	1%	1%
518	523	1	4	6.39-6.61	50%	52%	53%	52%	52%	52%	52%	51%	2%	2%	2%	1%	0%	3%	1%	1%
523	533	2	9	5.28-5.51	12%	26%	39%	55%	11%	27%	36%	55%	0%	0%	2%	2%	0%	0%	0%	1%
524	531	2	6	5.47-5.69	13%	34%	43%	54%	11%	33%	42%	51%	1%	0%	1%	1%	0%	1%	1%	1%
524	533	2	8	4.32-4.56	9%	26%	39%	58%	8%	24%	37%	57%	1%	1%	1%	1%	0%	1%	1%	2%
524	542	3	16	8.92-9.23	8%	18%	32%	54%	7%	17%	29%	51%	0%	1%	0%	1%	0%	0%	1%	1%
524	551	4	25	13.20-13.54	6%	15%	26%	44%	10%	18%	25%	43%	0%	0%	0%	1%	0%	0%	1%	1%
532	551	3	17	13.63-14.02	7%	15%	24%	41%	12%	19%	23%	41%	0%	0%	1%	1%	0%	0%	0%	0%
543	551	1	7	11.64-11.95	2%	7%	11%	18%	8%	12%	13%	25%	0%	0%	0%	1%	0%	1%	0%	0%
546	551	1	4	10.82-10.91	1%	1%	1%	1%	0%	1%	1%	9%	0%	0%	0%	0%	0%	0%	0%	1%
551	569	2	15	14.97-15.31	1%	4%	11%	15%	1%	4%	11%	14%	0%	0%	0%	1%	0%	0%	0%	1%
552	570	3	15	15.01-15.61	1%	4%	11%	15%	1%	3%	10%	14%	0%	0%	0%	0%	0%	0%	0%	1%
562	570	2	5	15.01-15.36	2%	3%	7%	11%	2%	2%	6%	10%	1%	1%	0%	1%	0%	1%	0%	1%
571	583	2	11	9.61-9.92	1%	2%	6%	15%	1%	1%	4%	11%	0%	0%	0%	1%	0%	0%	0%	0%
584	598	3	10	12.12-12.38	2%	10%	21%	32%	2%	9%	19%	29%	0%	1%	1%	1%	0%	0%	0%	1%
602	618	2	13	13.45-13.54	1%	3%	7%	8%	1%	3%	7%	8%	1%	0%	0%	1%	1%	0%	0%	0%
602	632	4	27	16.78-17.13	4%	8%	18%	23%	4%	8%	17%	23%	0%	0%	1%	1%	0%	0%	0%	1%

635	649	2	13	16.87-17.17	2%	8%	15%	18%	2%	8%	15%	18%	1%	1%	0%	0%	0%	0%	0%	0%
649	666	4	16	10.82-11.26	0%	0%	0%	1%	0%	0%	1%	1%	0%	0%	0%	0%	0%	0%	0%	0%
653	666	3	12	8.32-8.49	1%	1%	1%	2%	1%	0%	1%	2%	0%	0%	0%	1%	0%	0%	0%	0%
667	671	2	3	16.05-16.39	1%	0%	0%	0%	0%	0%	1%	0%	0%	0%	1%	1%	0%	0%	0%	0%
667	687	4	19	12.64-12.98	2%	4%	9%	15%	2%	4%	9%	15%	0%	0%	0%	1%	0%	0%	0%	0%
672	687	2	14	10.43-10.65	4%	7%	14%	24%	4%	7%	14%	23%	0%	0%	1%	2%	0%	0%	0%	0%
676	687	2	10	10.82-11.13	4%	7%	16%	29%	4%	8%	16%	29%	0%	0%	1%	3%	0%	0%	0%	1%
691	697	1	5	8.19-8.32	17%	29%	31%	33%	18%	29%	30%	32%	1%	0%	0%	0%	1%	0%	0%	0%
698	708	2	9	6.13-6.35	0%	1%	2%	2%	0%	1%	2%	2%	0%	0%	0%	0%	0%	0%	0%	0%
698	709	3	10	7.62-7.93	0%	0%	1%	1%	0%	0%	1%	1%	0%	0%	0%	0%	0%	0%	0%	0%
698	734	4	35	12.59-13.02	20%	24%	27%	32%	18%	24%	27%	31%	0%	0%	1%	1%	0%	0%	0%	1%
716	734	3	17	9.05-9.27	42%	50%	54%	61%	38%	51%	54%	60%	0%	0%	1%	1%	0%	0%	1%	1%
720	734	4	13	7.32-7.49	43%	51%	51%	51%	37%	51%	51%	51%	3%	1%	1%	1%	1%	1%	1%	1%
735	744	2	7	9.74-9.96	4%	20%	58%	63%	2%	17%	53%	62%	0%	2%	1%	3%	0%	0%	1%	2%
745	764	2	16	16.65-17.22	1%	2%	4%	15%	1%	1%	3%	14%	0%	0%	0%	1%	0%	0%	0%	1%
745	766	3	18	16.39-17.09	4%	6%	8%	18%	4%	6%	8%	17%	0%	0%	0%	0%	0%	0%	0%	1%
746	764	2	15	15.92-16.44	1%	1%	3%	15%	1%	1%	3%	14%	0%	0%	0%	1%	0%	0%	0%	0%
746	766	2	17	15.83-16.52	4%	7%	8%	19%	4%	7%	8%	18%	0%	0%	0%	1%	0%	0%	0%	0%
749	764	2	12	14.19-14.32	1%	2%	4%	16%	1%	2%	3%	15%	0%	0%	1%	1%	0%	0%	0%	1%
751	764	2	10	13.58-13.89	1%	2%	4%	15%	1%	1%	3%	14%	0%	0%	0%	1%	0%	0%	0%	0%
751	766	3	12	13.58-14.10	5%	9%	11%	20%	5%	9%	10%	18%	0%	0%	0%	1%	0%	0%	0%	0%
769	781	3	10	11.73-12.20	9%	16%	23%	25%	10%	15%	22%	23%	0%	0%	1%	1%	0%	0%	0%	1%
770	781	3	9	11.43-11.64	10%	15%	20%	21%	10%	15%	20%	20%	0%	0%	1%	1%	0%	0%	0%	1%
770	789	3	16	14.49-14.75	8%	16%	22%	22%	9%	15%	21%	22%	0%	1%	1%	2%	0%	1%	1%	1%
782	789	1	5	13.11-13.37	17%	30%	49%	50%	17%	29%	47%	47%	1%	1%	2%	3%	0%	1%	0%	2%
793	811	3	17	12.94-13.33	4%	13%	21%	27%	4%	12%	20%	25%	0%	0%	1%	1%	0%	1%	0%	1%
794	799	1	4	8.32-8.62	19%	30%	45%	46%	19%	34%	44%	44%	1%	2%	3%	5%	0%	1%	2%	2%
815	821	2	5	10.95-11.13	-1%	0%	0%	0%	0%	0%	0%	0%	1%	0%	0%	0%	0%	0%	0%	0%
815	830	2	14	15.66-15.87	4%	8%	13%	22%	5%	8%	12%	20%	0%	0%	1%	2%	0%	0%	0%	1%
815	831	2	15	16.82-17.09	3%	6%	11%	21%	3%	6%	10%	19%	0%	0%	1%	2%	0%	0%	0%	1%
831	839	1	6	14.45-14.71	1%	3%	8%	15%	1%	3%	9%	15%	0%	0%	1%	1%	0%	0%	0%	1%
840	844	1	3	6.97-7.23	4%	11%	26%	29%	5%	11%	26%	25%	0%	0%	2%	0%	0%	1%	1%	1%
847	858	3	10	9.96-10.09	10%	16%	22%	27%	9%	15%	23%	26%	1%	1%	1%	1%	0%	0%	0%	2%
847	859	3	11	9.48-9.70	9%	14%	23%	32%	9%	15%	22%	32%	0%	0%	1%	1%	0%	0%	0%	1%
848	858	1	9	8.75-8.92	10%	15%	24%	28%	9%	16%	23%	28%	1%	1%	0%	1%	1%	0%	0%	1%
849	858	2	8	8.71-8.92	8%	15%	23%	29%	8%	14%	23%	29%	0%	0%	1%	0%	0%	0%	1%	0%
859	893	4	33	11.56-11.95	21%	27%	31%	32%	20%	28%	30%	31%	0%	0%	1%	2%	0%	0%	1%	1%
873	879	2	5	3.53-3.76	3%	13%	27%	34%	3%	14%	26%	33%	0%	1%	2%	3%	0%	2%	1%	1%
880	893	2	12	8.88-9.10	9%	25%	31%	32%	9%	25%	30%	31%	0%	1%	1%	2%	0%	0%	0%	1%
897	903	1	5	4.97-5.06	1%	1%	1%	1%	2%	1%	1%	1%	0%	1%	0%	0%	0%	1%	1%	0%
909	920	2	10	10.87-11.08	3%	6%	11%	14%	3%	7%	11%	14%	0%	1%	1%	1%	0%	0%	1%	1%
909	922	2	12	12.59-12.85	1%	3%	6%	7%	1%	4%	6%	7%	0%	1%	0%	1%	0%	0%	0%	0%
911	921	2	9	11.86-11.99	2%	4%	9%	10%	2%	5%	9%	10%	0%	0%	1%	1%	0%	0%	1%	1%
922	929	1	6	7.62-7.75	1%	1%	5%	7%	1%	1%	5%	7%	0%	0%	0%	1%	0%	0%	0%	1%
923	929	1	5	6.30-6.57	0%	1%	4%	5%	0%	1%	3%	4%	0%	0%	1%	1%	0%	0%	0%	0%
930	956	4	24	11.73-11.99	5%	9%	17%	22%	5%	10%	17%	21%	0%	0%	1%	1%	0%	0%	1%	1%
957	961	1	3	12.38-12.55	0%	4%	5%	5%	0%	5%	5%	5%	0%	1%	1%	1%	0%	1%	0%	1%
961	976	2	14	7.93-8.19	28%	29%	41%	47%	27%	31%	41%	46%	2%	1%	1%	2%	0%	0%	0%	0%
961	980	4	18	10.39-10.61	15%	18%	26%	34%	15%	18%	26%	32%	0%	0%	1%	1%	0%	0%	0%	1%
962	980	3	17	9.48-9.70	13%	15%	25%	31%	13%	15%	24%	30%	0%	1%	1%	2%	0%	0%	1%	3%
989	997	2	7	7.62-7.84	4%	11%	26%	30%	4%	12%	26%	30%	0%	0%	1%	0%	0%	0%	1%	1%
990	997	1	6	5.51-5.74	6%	15%	33%	38%	5%	15%	32%	37%	0%	0%	2%	1%	0%	0%	1%	1%
990	997	2	6	5.51-5.74	6%	15%	33%	39%	5%	15%	32%	38%	0%	0%	2%	0%	0%	0%	2%	2%
1002	1006	1	3	15.57-15.70	-1%	0%	5%	27%	-1%	0%	6%	28%	0%	0%	0%	0%	0%	1%	0%	0%
1021	1028	1	6	9.40-9.53	1%	0%	1%	1%	1%	1%	1%	2%	1%	0%	0%	1%	1%	0%	1%	0%
1022	1033	2	10	6.13-6.30	11%	19%	27%	30%	11%	19%	26%	29%	0%	0%	0%	1%	1%	0%	0%	1%
1022	1035	3	12	6.79-6.97	9%	16%	26%	32%	9%	16%	24%	32%	0%	1%	0%	1%	0%	1%	0%	1%
1022	1036	3	13	8.92-9.18	7%	14%	22%	29%	7%	13%	21%	28%	0%	1%	0%	0%	0%	0%	0%	0%
1022	1037	3	14	8.97-9.23	8%	14%	22%	28%	7%	14%	21%	28%	0%	1%	0%	1%	0%	0%	0%	1%
1029	1035	1	5	3.53-3.81	16%	32%	52%	63%	17%	31%	51%	63%	2%	0%	1%	1%	0%	0%	1%	1%
1039	1055	2	15	8.92-9.10	17%	21%	24%	24%	17%	22%	23%	23%	0%	0%	1%	1%	0%	0%	0%	1%
1056	1068	3	11	12.33-12.42	56%	71%	75%	78%	57%	73%	77%	77%	0%	1%	3%	2%	1%	0%	1%	1%
1060	1068	2	7	3.76-4.09	52%	54%	56%	56%	52%	56%	56%	55%	0%	1%	1%	1%	0%	2%	0%	2%
