# Supplementary material for: Dynamic steps in receptor tyrosine kinase mediated activation of class IA phosphoinositide 3-kinases (PI3K) captured by H/D exchange (HDX-MS)
Source: Adv Biol Regul. 2013 Jan;53(1):97–110. doi: 10.1016/j.jbior.2012.09.005 (PMC3613897; doi:10.1016/j.jbior.2012.09.005)
Supplement: Supplementary file 3 [file mmc2.pdf]

					GLOBAL HDX LEVELS p110 beta / p85 alpha				p110 beta peptides p110 beta / p85 alpha + pY				p110 beta / p85 alpha				p110 beta / p85 alpha + pY			
Start	End	CS	#D	RT	3	30	300	3000	3	30	300	3000	3	30	300	3000	3	30	300	3000
42	51	2	7	9.84-9.93	4%	19%	30%	46%	3%	17%	28%	45%	0%	1%	1%	1%	0%	0%	1%	1%
42	54	2	10	12.00-12.13	6%	21%	29%	41%	5%	20%	28%	40%	0%	1%	1%	0%	0%	1%	0%	1%
55	59	1	3	8.72-8.81	0%	0%	0%	0%	0%	0%	0%	0%	0%	0%	0%	0%	0%	0%	1%	0%
60	69	2	7	12.17-12.34	11%	21%	30%	44%	11%	20%	30%	43%	0%	0%	1%	1%	0%	1%	0%	1%
73	79	1	5	13.68-13.77	1%	10%	27%	41%	1%	8%	24%	39%	0%	0%	0%	0%	0%	1%	0%	0%
80	88	1	7	9.93-10.06	0%	5%	18%	22%	0%	5%	18%	22%	0%	0%	0%	0%	0%	0%	0%	0%
89	101	2	11	11.09-11.26	9%	19%	28%	41%	8%	17%	28%	40%	0%	2%	1%	1%	0%	1%	0%	2%
93	106	2	11	12.51-12.82	1%	3%	14%	33%	1%	3%	13%	33%	0%	0%	1%	1%	0%	0%	0%	1%
102	111	2	6	14.24-14.33	1%	1%	1%	6%	0%	0%	1%	8%	0%	0%	0%	1%	0%	0%	0%	0%
107	111	1	2	10.70-10.88	0%	0%	1%	0%	1%	-1%	0%	0%	0%	0%	1%	0%	0%	1%	1%	0%
107	113	2	4	10.36-10.57	11%	22%	23%	22%	9%	22%	22%	22%	0%	1%	1%	1%	0%	1%	0%	1%
110	122	2	10	7.12-7.16	36%	41%	39%	38%	35%	41%	39%	38%	1%	2%	1%	1%	2%	3%	0%	1%
110	129	2	17	9.76-9.97	19%	25%	27%	34%	20%	26%	30%	37%	1%	2%	0%	0%	1%	2%	0%	0%
112	122	3	8	6.90-6.99	45%	49%	47%	45%	45%	49%	48%	46%	0%	2%	0%	0%	3%	2%	1%	1%
112	129	4	15	10.32-10.70	21%	27%	30%	39%	22%	29%	34%	43%	1%	2%	0%	0%	1%	2%	0%	0%
114	122	2	6	6.42-6.60	46%	47%	48%	47%	47%	48%	48%	47%	1%	2%	1%	1%	1%	0%	1%	1%
114	129	3	13	10.19-10.32	21%	26%	31%	41%	21%	28%	33%	45%	1%	2%	1%	0%	1%	2%	1%	0%
123	129	1	5	10.10-10.23	1%	4%	13%	38%	1%	6%	22%	51%	0%	0%	0%	1%	0%	1%	1%	0%
130	136	1	5	4.79-4.88	9%	15%	30%	37%	10%	21%	36%	37%	1%	2%	0%	1%	0%	1%	0%	1%
130	147	3	15	10.14-10.36	12%	23%	32%	36%	11%	23%	32%	35%	0%	2%	0%	0%	0%	1%	0%	0%
130	163	5	31	12.60-12.73	5%	12%	27%	41%	5%	13%	28%	40%	0%	1%	0%	0%	0%	1%	0%	0%
131	136	1	4	4.74-4.88	8%	15%	33%	40%	9%	21%	39%	40%	1%	3%	0%	0%	0%	2%	1%	1%
137	147	2	8	9.11-9.24	18%	35%	49%	55%	17%	34%	47%	54%	1%	2%	0%	1%	1%	1%	1%	1%
148	158	2	9	4.57-4.74	1%	5%	17%	27%	1%	5%	17%	27%	0%	1%	0%	1%	0%	1%	0%	0%
148	163	2	14	9.07-9.19	1%	7%	23%	34%	2%	8%	24%	33%	0%	1%	0%	0%	0%	0%	0%	0%
164	168	1	3	14.07-14.20	41%	75%	86%	86%	41%	79%	89%	88%	0%	0%	0%	0%	2%	0%	0%	0%
164	188	3	19	16.48-16.83	21%	28%	34%	44%	21%	28%	35%	45%	1%	2%	1%	1%	1%	2%	0%	1%
170	188	2	13	12.38-12.64	31%	37%	39%	50%	30%	37%	41%	51%	1%	2%	1%	1%	1%	2%	1%	1%
189	197	2	7	8.81-8.98	40%	58%	59%	57%	36%	57%	58%	57%	1%	2%	0%	0%	2%	2%	2%	1%
198	206	2	7	10.88-10.92	20%	24%	27%	27%	22%	26%	30%	31%	0%	1%	0%	0%	1%	1%	0%	0%
198	207	2	8	10.10-10.36	23%	27%	29%	30%	26%	30%	32%	34%	1%	2%	0%	1%	1%	1%	1%	1%
211	226	2	12	12.99-13.12	16%	26%	28%	32%	15%	25%	28%	32%	0%	1%	0%	1%	1%	2%	0%	0%
212	225	2	10	11.05-11.09	14%	26%	29%	35%	13%	26%	29%	34%	1%	1%	0%	0%	0%	1%	0%	0%
212	226	2	11	12.77-12.94	12%	22%	25%	30%	11%	22%	24%	29%	1%	2%	0%	0%	1%	1%	0%	0%
226	245	2	17	8.59-8.72	19%	31%	36%	39%	17%	31%	37%	38%	0%	1%	0%	0%	1%	2%	1%	1%
227	245	2	16	7.60-7.68	20%	33%	38%	41%	19%	33%	38%	40%	1%	2%	0%	0%	1%	2%	1%	1%
249	265	3	14	12.34-12.69	8%	12%	20%	27%	8%	12%	20%	27%	1%	2%	1%	1%	1%	1%	0%	0%
249	267	2	16	13.77-13.94	10%	16%	23%	30%	10%	16%	24%	31%	0%	1%	0%	0%	1%	1%	0%	1%
267	274	1	6	9.97-10.19	1%	1%	3%	14%	1%	1%	2%	13%	0%	0%	0%	0%	0%	0%	0%	0%
275	281	2	4	9.41-9.58	6%	26%	39%	48%	5%	25%	38%	47%	0%	0%	0%	1%	0%	0%	0%	1%
275	283	2	6	12.64-12.99	5%	16%	18%	21%	4%	15%	18%	21%	0%	0%	0%	0%	0%	0%	0%	0%
284	297	3	12	9.41-9.63	2%	13%	42%	61%	2%	13%	44%	61%	0%	0%	1%	0%	0%	1%	0%	0%
287	297	2	9	7.64-7.94	2%	14%	48%	72%	1%	14%	51%	70%	0%	1%	1%	1%	0%	0%	2%	1%
302	334	4	26	11.14-11.31	29%	39%	40%	41%	29%	39%	40%	41%	1%	3%	0%	0%	2%	2%	0%	0%
335	347	2	11	8.12-8.33	30%	37%	36%	36%	30%	37%	37%	37%	0%	1%	0%	0%	2%	1%	0%	1%
338	347	2	8	4.39-4.70	47%	55%	56%	57%	45%	55%	56%	58%	1%	1%	1%	0%	2%	0%	0%	1%
338	348	2	9	4.57-4.79	48%	54%	56%	55%	48%	54%	54%	56%	1%	1%	0%	0%	2%	1%	2%	1%
364	371	2	6	7.21-7.42	3%	9%	26%	45%	2%	8%	24%	44%	0%	1%	1%	2%	0%	1%	0%	0%
364	372	1	7	7.55-7.77	6%	17%	30%	41%	5%	17%	28%	41%	0%	1%	0%	1%	0%	1%	0%	1%
364	387	2	21	13.51-13.72	4%	8%	11%	14%	4%	7%	10%	14%	0%	2%	0%	0%	0%	1%	0%	0%
373	387	2	12	13.68-13.90	1%	4%	6%	8%	1%	3%	6%	8%	0%	1%	1%	1%	0%	1%	0%	1%
388	394	1	5	14.84-15.10	1%	1%	4%	17%	1%	1%	4%	17%	0%	0%	0%	0%	0%	0%	0%	0%
388	397	1	7	14.11-14.37	1%	1%	5%	23%	1%	1%	5%	24%	0%	1%	0%	0%	0%	0%	0%	0%
388	402	2	12	16.83-16.92	0%	0%	3%	15%	0%	0%	3%	14%	0%	0%	0%	2%	0%	0%	0%	1%
405	443	4	35	8.38-8.59	22%	23%	25%	26%	23%	25%	25%	26%	0%	2%	0%	0%	1%	2%	0%	0%
406	443	4	34	8.03-8.42	24%	26%	26%	25%	25%	26%	27%	26%	0%	2%	1%	0%	2%	1%	1%	1%
409	443	3	31	7.90-8.33	23%	25%	22%	22%	23%	24%	23%	23%	1%	2%	0%	0%	3%	1%	0%	1%
444	456	3	11	11.57-11.82	4%	12%	21%	27%	3%	11%	21%	27%	0%	2%	2%	1%	0%	1%	0%	1%
444	459	2	14	14.63-14.93	2%	10%	20%	25%	2%	10%	19%	24%	0%	3%	1%	1%	0%	1%	1%	1%
449	456	1	6	3.67-3.72	6%	16%	33%	35%	4%	13%	33%	37%	0%	1%	1%	0%	0%	1%	1%	1%
457	465	2	7	13.81-14.07	23%	27%	24%	24%	25%	27%	25%	24%	1%	0%	0%	0%	1%	2%	0%	0%
460	469	2	7	13.46-13.59	29%	41%	37%	35%	29%	39%	36%	35%	1%	3%	1%	0%	1%	3%	0%	0%
470	478	1	6	11.61-11.87	2%	13%	34%	47%	3%	13%	38%	49%	1%	1%	0%	1%	0%	0%	1%	0%
470	488	2	15	12.69-12.90	16%	25%	42%	58%	16%	26%	45%	60%	0%	0%	0%	1%	0%	1%	0%	0%
492	513	3	16	11.39-11.39	14%	19%	27%	41%	15%	20%	30%	43%	0%	1%	0%	0%	1%	1%	0%	0%
492	517	3	20	11.52-11.82	11%	18%	37%	51%	12%	20%	40%	52%	0%	1%	0%	1%	1%	2%	1%	0%
510	517	1	6	4.26-4.39	2%	10%	49%	66%	2%	12%	54%	67%	0%	1%	1%	1%	0%	1%	0%	2%
518	525	1	6	3.53-3.86	73%	72%	73%	74%	73%	74%	75%	74%	1%	0%	0%	1%	2%	0%	1%	0%
518	538	3	18	10.79-11.01	36%	44%	42%	40%	36%	43%	42%	41%	1%	3%	0%	0%	2%	2%	0%	0%
518	540	2	20	8.72-9.11	34%	44%	47%	46%	34%	44%	47%	46%	1%	2%	0%	0%	1%	2%	1%	0%
518	540	4	20	8.81-8.98	35%	45%	48%	48%	34%	44%	48%	47%	1%	2%	0%	1%	2%	2%	0%	1%
518	550	4	29	14.54-15.02	20%	37%	43%	46%	19%	33%	45%	48%	1%	1%	0%	0%	1%	1%	0%	0%
538	549	2	9	12.08-12.43	18%	42%	48%	54%	15%	35%	50%	56%	0%	1%	0%	0%	0%	1%	0%	2%
539	549	2	8	9.32-9.54	18%	40%	46%	55%	15%	31%	50%	58%	0%	2%	1%	0%	0%	2%	1%	0%
539	550	3	9	12.13-12.43	17%	41%	47%	54%	15%	35%	51%	57%	0%	1%	0%	0%	0%	0%	0%	1%
541	550	2	7	12.82-13.07	22%	52%	58%	58%	18%	44%	58%	56%	1%	1%	0%	0%	1%	2%	0%	1%
543	550	2	5	11.74-11.91	26%	60%	70%	70%	22%	52%	69%	69%	2%	1%	1%	0%	1%	1%	0%	1%
550	554	1	3	5.23-5.32	13%	31%	35%	46%	35%	41%	56%	54%	0%	2%	0%	1%	1%	1%	1%	0%
550	556	1	5	8.03-8.20	3%	10%	10%	21%	10%	18%	32%	42%	0%	1%	0%	3%	2%	2%	0%	2%
551	556	1	4	5.68-5.77	4%	6%	10%	27%	8%	15%	41%	50%	0%	0%	1%	0%	0%	2%	1%	1%
556	565	2	8	14.97-15.10	0%	5%	18%	28%	1%	8%	38%	77%	0%	1%	1%	1%	0%	1%	3%	0%
557	561	1	3	16.92-17.22	0%	0%	1%	7%	1%	3%	29%	78%	0%	0%	1%	1%	0%	0%	1%	1%
557	565	2	7	13.33-13.46	0%	5%	18%	27%	1%	10%	39%	68%	0%	0%	0%	1%	0%	1%	0%	1%
557	578	3	18	16.57-16.61	2%	7%	16%	25%	3%	10%	28%	44%	0%	1%	0%	0%	0%	1%	0%	0%
558	565	2	6	11.91-12.04	0%	6%	20%	31%	1%	10%	42%	65%	0%	1%	1%	2%	1%	1%	1%	0%
558	578	3	17	16.05-16.22	2%	7%	17%	26%	3%	10%	31%	45%	0%	1%	0%	0%	0%	1%	1%	1%
561	565	1	3	4.12-4.30	1%	10%	43%	57%	3%	17%	56%	57%	0%	1%	1%	1%	0%	2%	0%	1%
566	578	2	9	14.50-14.63	3%	6%	11%	17%	3%	9%	22%	38%	0%	1%	0%	0%	0%	1%	1%	0%
568	578	2	7	16.61-17.00	4%	9%	8%	14%	4%	12%	20%	31%	0%	0%	0%	0%	0%	1%	0%	0%
577	591	2	13	13.94-14.11	1%	6%	13%	23%	1%	6%	16%	26%	0%	1%	0%	1%	0%	1%	0%	3%
579	591	3	11	12.34-12.64	1%	6%	16%	28%	1%	6%	17%	30%	0%	0%	0%	0%	0%	0%	1%	0%
595	607	3	8	12.86-13.03	12%	14%	22%	34%	11%	14%	22%	34%	0%	0%	0%	0%	0%	1%	0%	0%
595	609	3	10	14.11-14.28	9%	11%	17%	26%	9%	11%	18%	27%	0%	0%	0%	0%	0%	0%	0%	1%
622	627	1	4	12.21-12.38	0%	0%	1%	0%	0%	0%	0%	1%	0%	0%	0%	0%	0%	0%	0%	0%
627	632	1	4	5.28-5.37	43%	51%	54%	68%	41%	50%	53%	67%	1%	3%	2%	4%	1%	1%	0%	1%
628	634	1	5	4.61-4.83	24%	32%	44%	52%	23%	32%	44%	51%	1%	2%	2%	1%	1%	1%	1%	0%
643	652	2	7	14.41-14.50	0%	0%	1%	4%	0%	0%	0%	4%	0%	0%	0%	0%	0%	0%	0%	0%
643	653	2	8	13.51-13.77	0%	0%	2%	8%	0%	0%	1%	8%	0%	0%	0%	0%	0%	0%	0%	1%
674	678	2	2	15.53-15.66	1%	0%	0%	1%	0%	0%	0%	0%	1%	0%	1%	0%	0%	0%	1%	3%
674	688	3	12	12.34-12.64	8%	9%	10%	20%	8%	9%	10%	19%	0%	1%	1%	1%	0%	1%	0%	0%
675	688	2	11	11.48-11.69	8%	10%	11%	22%	8%	10%	12%	22%	1%	1%	0%	0%	1%	1%	0%	0%
675	691	2	14	13.51-13.72	7%	9%	16%	29%	6%	8%	15%	28%	0%	1%	0%	1%	0%	0%	0%	0%
681	691	2	8	13.03-13.29	11%	15%	27%	49%	10%	13%	26%	47%	1%	1%	1%	1%	1%	1%	0%	0%
696	716	3	19	9.89-9.93	3%	8%	13%	18%	3%	9%	14%	22%	0%	1%	0%	0%	1%	1%	0%	0%
698	706	1	7	6.25-6.34	10%	24%	31%	32%	10%	24%	32%	32%	1%	1%	0%	1%	2%	1%	1%	0%
698	716	2	17	9.24-9.45	2%	6%	10%	15%	2%	7%	11%	17%	0%	1%	0%	0%	0%	1%	0%	0%
698	725	6	26	10.83-11.05	1%	4%	12%	22%	2%	4%	12%	22%	0%	0%	0%	1%	0%	0%	0%	0%
707	714	1	6	4.39-4.70	1%	1%	3%	13%	1%	1%	4%	15%	0%	1%	0%	0%	0%	0%	0%	0%
715	725	1	9	9.80-9.97	2%	5%	22%	39%	3%	6%	26%	41%	0%	0%	0%	0%	0%	0%	1%	1%
717	725	2	7	8.59-8.76	2%	7%	29%	51%	3%	8%	32%	53%	0%	0%	0%	0%	1%	1%	1%	1%
726	730	1	3	6.99-7.16	35%	65%	84%	86%	33%	66%	85%	86%	1%	3%	0%	0%	1%	1%	1%	1%
726	742	4	15	6.73-6.90	39%	49%	49%	48%	38%	50%	50%	48%	1%	2%	0%	0%	2%	2%	1%	2%
726	746	3	19	7.90-8.16	26%	33%	36%	37%	26%	34%	36%	37%	1%	2%	1%	0%	1%	2%	0%	2%
731	742	3	10	4.17-4.39	46%	56%	54%	52%	46%	56%	55%	54%	1%	3%	1%	0%	3%	2%	0%	2%
731	746	2	14	6.12-6.21	25%	32%	35%	38%	25%	33%	35%	37%	1%	3%	0%	1%	1%	1%	0%	2%
743	750	2	6	3.90-4.17	29%	31%	48%	61%	30%	32%	47%	61%	1%	1%	1%	0%	1%	0%	1%	1%
747	768	2	18	14.84-15.15	3%	12%	32%	61%	3%	12%	31%	60%	0%	0%	0%	1%	0%	0%	0%	1%
751	768	2	14	15.40-15.71	2%	9%	21%	49%	2%	8%	22%	48%	0%	0%	0%	1%	0%	1%	0%	0%
771	787	4	14	11.57-11.87	15%	24%	32%	42%	15%	25%	33%	40%	0%	2%	0%	0%	1%	2%	0%	0%
772	787	3	13	11.14-11.22	12%	18%	28%	38%	13%	18%	28%	36%	1%	2%	0%	1%	1%	0%	0%	0%
788	798	2	9	10.70-10.92	43%	49%	51%	54%	42%	48%	50%	53%	2%	2%	0%	0%	2%	2%	1%	0%
788	801	2	12	11.18-11.35	35%	45%	51%	54%	35%	43%	51%	53%	2%	2%	2%	1%	2%	1%	1%	1%
799	814	3	14	11.69-11.87	4%	19%	32%	35%	4%	20%	33%	36%	0%	1%	1%	1%	0%	1%	1%	2%
802	814	2	11	10.49-10.79	5%	20%	33%	36%	5%	21%	34%	36%	0%	0%	1%	1%	0%	0%	0%	1%
815	819	1	3	12.25-12.30	0%	-1%	0%	0%	0%	0%	1%	2%	0%	0%	0%	0%	0%	0%	0%	0%
820	826	2	5	16.09-16.09	-4%	-5%	-4%	-3%	-5%	-5%	-3%	-5%	1%	0%	2%	1%	0%	1%	0%	1%
826	834	2	7	13.29-13.59	0%	3%	7%	12%	0%	2%	7%	13%	0%	0%	0%	1%	0%	1%	0%	0%
827	834	2	6	12.38-12.51	1%	3%	11%	16%	1%	4%	11%	18%	0%	0%	0%	0%	0%	0%	0%	1%
834	842	1	6	14.33-14.54	1%	7%	22%	29%	3%	8%	26%	32%	0%	1%	0%	1%	0%	0%	1%	1%
835	842	1	5	13.20-13.55	2%	9%	28%	37%	3%	10%	32%	40%	1%	1%	1%	2%	0%	0%	0%	0%
843	850	2	6	5.60-5.73	14%	18%	24%	36%	17%	19%	27%	36%	0%	1%	1%	0%	1%	0%	0%	0%
851	859	1	7	7.86-8.38	24%	36%	48%	60%	26%	39%	61%	69%	0%	1%	0%	0%	1%	1%	0%	0%
853	859	1	5	4.61-4.83	31%	38%	47%	63%	35%	42%	62%	72%	1%	1%	1%	2%	1%	1%	0%	1%
866	875	1	8	5.32-5.64	80%	80%	79%	78%	79%	80%	79%	76%	2%	1%	0%	0%	1%	0%	0%	1%
877	881	1	3	5.55-5.82	19%	57%	74%	73%	25%	69%	74%	72%	0%	2%	0%	1%	0%	3%	0%	1%
882	894	2	11	13.77-13.90	10%	23%	25%	29%	10%	22%	28%	33%	0%	1%	0%	0%	0%	2%	0%	1%
883	894	1	10	12.73-13.03	10%	26%	29%	32%	10%	25%	30%	36%	1%	1%	1%	0%	0%	2%	0%	1%
895	900	1	4	5.10-5.28	1%	0%	1%	5%	1%	1%	2%	7%	0%	0%	0%	0%	0%	0%	0%	0%
902	907	1	4	6.51-6.69	0%	0%	0%	0%	0%	0%	0%	1%	0%	0%	0%	0%	0%	0%	0%	0%
908	912	1	3	6.69-6.77	0%	0%	0%	0%	0%	0%	0%	0%	0%	0%	0%	0%	0%	1%	0%	0%
908	926	3	17	14.37-14.46	2%	4%	6%	7%	2%	5%	7%	9%	0%	0%	0%	0%	0%	0%	0%	0%
925	945	4	19	15.19-15.53	6%	10%	13%	22%	7%	11%	16%	25%	0%	1%	0%	0%	0%	0%	0%	0%
926	945	4	18	15.19-15.36	5%	11%	13%	22%	7%	11%	16%	25%	0%	0%	0%	1%	0%	1%	1%	0%
927	933	1	5	4.26-4.43	1%	2%	12%	20%	1%	3%	15%	26%	0%	0%	0%	0%	0%	0%	0%	0%
927	945	3	17	15.10-15.32	7%	12%	15%	24%	7%	13%	17%	27%	0%	1%	1%	0%	0%	1%	0%	0%
934	945	3	10	16.18-16.48	10%	18%	23%	39%	11%	19%	27%	42%	0%	1%	0%	1%	0%	2%	0%	0%
935	945	2	9	14.71-15.02	12%	20%	26%	43%	13%	22%	30%	46%	0%	1%	0%	0%	1%	1%	0%	1%
946	961	3	13	9.80-10.01	22%	30%	35%	47%	23%	35%	48%	54%	1%	2%	1%	0%	1%	2%	0%	0%
962	982	4	19	12.00-12.17	15%	23%	24%	26%	16%	23%	28%	34%	1%	2%	1%	0%	1%	2%	0%	1%
962	987	5	24	11.35-11.65	8%	12%	14%	16%	9%	13%	17%	23%	0%	2%	1%	0%	1%	1%	0%	0%
964	982	4	17	9.63-9.84	16%	23%	23%	25%	17%	23%	27%	29%	1%	2%	1%	0%	1%	2%	0%	0%
965	982	3	16	8.63-8.76	19%	26%	27%	29%	19%	26%	31%	32%	2%	1%	1%	1%	2%	2%	1%	0%
983	987	1	3	3.95-4.03	1%	2%	1%	1%	1%	1%	2%	1%	0%	0%	0%	0%	0%	0%	0%	0%
983	988	1	4	4.17-4.35	1%	0%	0%	0%	0%	0%	0%	0%	0%	0%	0%	0%	1%	0%	0%	0%
983	989	1	4	4.70-4.83	0%	0%	0%	0%	0%	0%	0%	1%	0%	0%	0%	0%	1%	0%	0%	0%
991	999	3	7	6.73-6.90	1%	3%	8%	13%	1%	3%	8%	13%	0%	0%	0%	0%	0%	1%	0%	0%
992	1000	2	7	8.50-8.72	1%	2%	6%	10%	1%	3%	5%	11%	0%	0%	0%	0%	0%	1%	1%	1%
1000	1004	1	3	17.18-17.35	0%	0%	0%	0%	0%	0%	0%	0%	0%	1%	0%	0%	0%	0%	0%	0%
1004	1008	1	3	16.48-16.70	0%	0%	0%	3%	0%	0%	1%	4%	0%	0%	0%	0%	0%	0%	0%	1%
1008	1015	1	5	13.42-13.55	3%	16%	34%	66%	2%	16%	35%	63%	0%	0%	1%	2%	0%	0%	0%	0%
1008	1026	3	16	12.86-13.25	11%	24%	34%	53%	11%	24%	36%	53%	0%	1%	0%	0%	0%	0%	0%	0%
1008	1049	5	39	15.88-16.09	10%	17%	25%	34%	10%	17%	26%	39%	0%	0%	0%	0%	0%	1%	0%	0%
1016	1026	3	9	8.94-9.11	15%	28%	37%	54%	15%	30%	39%	55%	0%	1%	1%	1%	1%	0%	0%	0%
1019	1026	1	6	8.81-9.11	45%	67%	66%	66%	42%	65%	66%	66%	1%	2%	1%	0%	2%	3%	0%	1%
1027	1035	1	7	7.38-7.64	31%	41%	42%	47%	28%	39%	42%	47%	0%	1%	1%	1%	2%	1%	2%	1%
1027	1048	4	20	13.03-13.55	9%	12%	19%	27%	8%	12%	21%	32%	0%	1%	0%	0%	1%	0%	0%	0%
1027	1049	3	21	14.41-14.59	8%	12%	18%	25%	7%	11%	20%	35%	0%	1%	0%	0%	0%	0%	0%	0%
1027	1059	4	31	15.79-15.96	9%	18%	30%	37%	21%	29%	37%	48%	0%	1%	0%	1%	1%	2%	0%	1%
1036	1048	2	11	9.24-9.41	1%	1%	6%	13%	1%	1%	8%	24%	0%	0%	0%	0%	0%	0%	0%	1%
1050	1059	2	8	11.52-11.65	9%	32%	62%	64%	52%	67%	66%	65%	0%	2%	0%	0%	1%	1%	0%	0%
1054	1059	1	4	9.54-9.71	3%	30%	72%	74%	53%	78%	77%	74%	1%	0%	0%	2%	3%	2%	0%	0%
1060	1070	2	9	3.90-4.12	51%	51%	51%	51%	49%	51%	52%	50%	1%	2%	1%	1%	2%	1%	0%	0%
