# Supplementary material for: Dynamic steps in receptor tyrosine kinase mediated activation of class IA phosphoinositide 3-kinases (PI3K) captured by H/D exchange (HDX-MS)
Source: Adv Biol Regul. 2013 Jan;53(1):97–110. doi: 10.1016/j.jbior.2012.09.005 (PMC3613897; doi:10.1016/j.jbior.2012.09.005)
Supplement: Supplementary file 4 [file mmc3.pdf]

					GLOBAL HDX LEVELS				p110 delta peptides				STDEVS							
					p110 delta / p85 alpha				p110 delta / p85 alpha + pY				p110 delta / p85 alpha				p110 delta / p85 alpha + pY			
Start	End	CS	#D	RT	3	30	300	3000	3	30	300	3000	3	30	300	3000	3	30	300	3000
12	20	1	7	6.88-7.18	50%	52%	58%	61%	48%	52%	58%	58%	1%	0%	4%	1%	0%	0%	2%	3%
24	42	2	15	15.93-16.24	10%	18%	24%	26%	8%	19%	23%	24%	1%	0%	2%	0%	1%	0%	1%	1%
43	59	3	14	13.56-13.95	4%	10%	20%	29%	3%	10%	20%	28%	0%	0%	1%	1%	0%	0%	0%	1%
60	67	1	5	7.40-7.40	27%	46%	59%	60%	23%	46%	58%	55%	1%	1%	4%	1%	1%	0%	1%	4%
71	91	3	19	11.19-11.49	18%	36%	44%	61%	16%	35%	43%	59%	0%	1%	1%	0%	1%	0%	0%	1%
86	96	2	8	12.57-12.57	2%	14%	27%	45%	1%	13%	25%	42%	1%	1%	1%	1%	0%	0%	1%	2%
102	120	2	17	11.93-12.23	38%	43%	46%	52%	36%	43%	46%	51%	0%	1%	1%	0%	1%	1%	1%	1%
120	127	2	6	6.44-6.75	6%	13%	31%	36%	6%	14%	30%	31%	1%	0%	3%	2%	0%	0%	0%	4%
120	138	2	16	13.00-13.30	10%	21%	31%	32%	9%	22%	32%	31%	0%	0%	2%	0%	0%	0%	1%	1%
120	139	3	17	14.30-14.60	9%	18%	27%	27%	8%	19%	27%	27%	0%	1%	2%	0%	0%	0%	1%	1%
140	146	2	5	8.52-8.74	1%	11%	27%	49%	2%	10%	26%	45%	0%	0%	1%	1%	0%	0%	0%	3%
146	162	2	15	13.61-13.99	9%	23%	48%	59%	7%	22%	46%	58%	1%	1%	2%	0%	0%	0%	1%	1%
162	191	3	24	16.54-16.93	36%	46%	55%	54%	35%	46%	55%	52%	0%	2%	2%	0%	0%	0%	1%	2%
178	191	3	10	13.26-13.56	50%	61%	69%	65%	48%	61%	70%	64%	0%	3%	3%	0%	1%	1%	1%	3%
192	202	2	9	7.83-8.05	32%	42%	50%	53%	29%	42%	50%	53%	1%	0%	2%	1%	1%	0%	1%	1%
201	216	2	13	13.99-14.30	20%	29%	40%	46%	17%	29%	40%	43%	1%	0%	2%	0%	1%	0%	1%	1%
217	238	3	18	8.61-8.82	35%	47%	53%	55%	31%	47%	53%	54%	1%	1%	2%	0%	1%	0%	1%	1%
228	238	2	7	8.74-9.04	51%	64%	71%	77%	46%	63%	71%	74%	1%	1%	1%	2%	2%	0%	0%	1%
239	250	1	10	10.16-10.46	3%	3%	4%	7%	2%	4%	4%	7%	0%	0%	1%	0%	0%	0%	0%	1%
251	259	1	6	15.24-15.55	3%	3%	3%	3%	3%	4%	4%	3%	0%	0%	1%	0%	0%	0%	0%	0%
259	266	1	6	15.80-15.80	7%	9%	9%	14%	6%	10%	9%	13%	0%	1%	1%	0%	0%	0%	1%	1%
265	283	3	16	11.02-11.32	4%	4%	5%	10%	4%	5%	5%	9%	0%	0%	1%	0%	0%	0%	0%	0%
283	315	3	23	6.88-7.18	58%	61%	61%	58%	56%	62%	62%	56%	0%	2%	3%	0%	1%	0%	1%	2%
317	327	2	9	16.97-17.27	9%	14%	23%	26%	7%	14%	22%	25%	0%	0%	1%	0%	0%	0%	1%	1%
328	337	2	8	4.77-4.99	9%	25%	39%	45%	8%	30%	40%	43%	0%	0%	3%	0%	1%	0%	1%	3%
328	341	4	12	7.79-8.00	9%	20%	27%	36%	7%	22%	28%	37%	1%	0%	2%	1%	1%	0%	1%	2%
342	354	2	11	12.96-13.26	3%	8%	9%	10%	4%	9%	9%	8%	0%	0%	1%	0%	0%	0%	0%	1%
354	362	1	7	4.86-5.08	18%	35%	41%	41%	14%	35%	42%	37%	1%	0%	4%	0%	1%	0%	1%	3%
355	377	3	20	12.57-12.87	16%	26%	35%	42%	14%	26%	35%	41%	0%	0%	1%	1%	1%	0%	1%	1%
366	377	2	9	12.57-12.87	11%	19%	27%	40%	9%	18%	26%	38%	0%	1%	2%	0%	1%	0%	1%	2%
378	387	1	7	13.95-14.25	0%	0%	8%	26%	0%	2%	6%	25%	0%	0%	0%	0%	0%	1%	1%	2%
378	392	2	12	16.67-17.67	0%	0%	4%	12%	0%	0%	3%	12%	0%	0%	0%	0%	0%	0%	0%	1%
388	392	1	3	11.41-11.62	0%	-1%	3%	1%	0%	1%	2%	2%	1%	0%	2%	2%	1%	1%	0%	3%
395	423	3	26	8.99-9.30	15%	17%	19%	19%	14%	17%	19%	19%	1%	1%	1%	1%	1%	1%	1%	0%
424	439	3	14	12.18-12.49	12%	21%	25%	30%	10%	20%	25%	29%	1%	1%	1%	0%	0%	0%	1%	1%
439	452	2	10	14.64-15.03	28%	33%	37%	34%	26%	34%	37%	34%	0%	2%	2%	0%	1%	0%	1%	1%
440	452	1	9	13.69-13.99	33%	39%	45%	42%	32%	41%	46%	42%	0%	2%	2%	0%	1%	0%	2%	1%
453	468	2	12	6.40-6.62	32%	52%	65%	72%	28%	53%	67%	71%	1%	0%	3%	1%	1%	0%	1%	4%
453	471	2	15	8.52-8.91	26%	42%	52%	57%	23%	43%	53%	56%	1%	1%	3%	1%	1%	0%	1%	3%
472	488	2	11	15.07-15.37	22%	34%	47%	53%	20%	34%	46%	50%	1%	1%	2%	0%	1%	0%	1%	1%
476	488	2	8	12.18-12.49	24%	39%	56%	64%	21%	38%	55%	62%	0%	1%	2%	0%	1%	0%	1%	2%
489	500	2	10	8.17-8.39	7%	12%	21%	33%	7%	14%	23%	33%	0%	0%	1%	0%	0%	0%	0%	2%
489	508	3	18	8.65-8.86	16%	19%	30%	37%	14%	21%	30%	37%	1%	1%	2%	1%	1%	0%	1%	1%
494	499	1	4	3.30-3.48	12%	19%	18%	16%	10%	21%	19%	13%	1%	1%	3%	0%	1%	1%	1%	3%
494	500	2	5	3.57-3.90	18%	21%	22%	20%	18%	23%	23%	17%	0%	0%	3%	1%	1%	0%	1%	3%
501	506	1	4	4.81-5.03	42%	51%	64%	62%	38%	52%	64%	58%	1%	1%	5%	1%	1%	1%	1%	2%
501	508	2	6	4.99-5.21	23%	29%	47%	56%	20%	31%	46%	51%	1%	0%	4%	1%	1%	1%	1%	3%
512	523	3	10	7.35-7.57	27%	32%	33%	34%	22%	32%	33%	30%	0%	0%	3%	0%	1%	0%	1%	2%
516	523	1	6	4.77-4.99	49%	50%	51%	47%	43%	52%	51%	44%	1%	1%	4%	2%	1%	1%	1%	2%
524	529	2	3	3.30-3.62	1%	0%	1%	10%	0%	9%	26%	28%	1%	0%	0%	1%	0%	3%	0%	3%
524	546	3	20	12.70-13.74	1%	5%	8%	11%	1%	5%	10%	13%	0%	0%	0%	0%	0%	0%	0%	1%
550	565	3	14	11.67-11.97	0%	1%	3%	4%	0%	1%	2%	3%	0%	0%	0%	0%	0%	0%	0%	0%
568	574	1	4	15.72-16.11	4%	20%	40%	65%	3%	20%	40%	61%	0%	1%	3%	1%	0%	0%	1%	1%
575	579	1	3	8.05-8.26	-1%	15%	60%	84%	-1%	12%	58%	91%	0%	1%	2%	0%	0%	1%	2%	10%
583	595	1	10	15.59-15.98	5%	17%	33%	37%	5%	17%	31%	32%	0%	0%	3%	1%	1%	1%	2%	1%
585	595	1	8	13.82-14.81	3%	11%	26%	30%	2%	10%	23%	27%	0%	0%	4%	2%	0%	0%	1%	2%
596	608	2	11	9.38-9.60	6%	10%	17%	22%	5%	10%	16%	21%	0%	0%	1%	1%	0%	0%	0%	1%
647	651	2	3	15.03-15.67	0%	1%	1%	0%	0%	1%	1%	1%	0%	0%	0%	0%	0%	0%	0%	0%
656	661	1	3	5.48-5.79	25%	27%	38%	65%	25%	26%	37%	65%	0%	0%	1%	1%	0%	0%	1%	1%
662	668	2	5	15.46-15.85	0%	0%	0%	0%	1%	0%	1%	0%	0%	0%	0%	0%	0%	0%	0%	0%
669	682	2	12	6.66-6.66	1%	3%	8%	9%	1%	4%	8%	9%	0%	1%	0%	1%	0%	1%	0%	0%
671	689	3	17	7.40-7.70	1%	3%	8%	13%	1%	3%	8%	12%	0%	0%	0%	0%	0%	0%	0%	0%
684	697	2	12	8.13-8.35	1%	2%	3%	9%	1%	2%	3%	8%	0%	0%	0%	1%	0%	0%	0%	0%
690	697	2	6	5.44-5.75	1%	1%	1%	2%	1%	1%	1%	2%	0%	0%	0%	0%	0%	0%	0%	0%
698	713	3	12	5.53-5.83	22%	39%	47%	51%	19%	38%	47%	48%	1%	1%	3%	1%	0%	0%	1%	2%
698	717	4	16	8.61-8.82	12%	19%	24%	32%	10%	20%	24%	29%	0%	1%	2%	0%	0%	0%	1%	2%
714	719	2	4	12.18-12.74	0%	0%	2%	1%	0%	1%	1%	1%	1%	1%	3%	0%	0%	2%	0%	0%
718	723	1	4	3.81-4.50	44%	47%	56%	66%	41%	46%	56%	64%	0%	3%	3%	2%	0%	1%	1%	1%
720	727	1	6	8.69-8.82	24%	45%	64%	73%	20%	43%	63%	71%	1%	0%	3%	2%	0%	1%	1%	3%
728	740	2	9	11.67-11.97	2%	12%	29%	31%	2%	11%	29%	30%	0%	0%	1%	0%	0%	0%	0%	0%
746	751	1	4	10.59-10.80	1%	6%	26%	40%	1%	5%	25%	38%	0%	0%	1%	0%	0%	0%	0%	2%
752	762	2	8	14.47-14.51	9%	10%	12%	18%	8%	11%	12%	17%	0%	1%	1%	0%	1%	0%	1%	1%
753	762	2	7	14.38-14.68	9%	11%	13%	20%	9%	11%	12%	18%	0%	0%	0%	0%	0%	0%	0%	1%
761	766	1	4	6.96-7.87	27%	31%	46%	44%	25%	32%	44%	41%	0%	1%	4%	1%	0%	0%	1%	5%
763	788	3	24	12.74-13.05	12%	21%	27%	28%	10%	22%	26%	27%	1%	0%	3%	2%	1%	0%	1%	2%
767	788	3	20	12.40-12.62	8%	18%	25%	28%	6%	18%	24%	26%	0%	0%	2%	1%	0%	0%	1%	1%
797	807	2	9	12.57-12.87	0%	3%	10%	10%	1%	3%	9%	10%	0%	0%	1%	0%	0%	0%	0%	0%
797	808	2	10	13.82-14.81	0%	2%	6%	6%	0%	1%	6%	5%	0%	0%	1%	0%	0%	0%	0%	1%
801	807	1	5	8.26-8.56	2%	7%	22%	24%	1%	6%	21%	22%	0%	0%	2%	0%	0%	0%	1%	3%
808	824	3	13	11.93-12.23	2%	4%	7%	13%	2%	4%	7%	12%	0%	0%	1%	0%	0%	0%	0%	1%
808	826	2	15	12.23-12.53	2%	4%	6%	12%	2%	4%	6%	11%	0%	0%	1%	0%	0%	0%	0%	0%
825	829	1	3	12.53-12.83	9%	41%	68%	68%	7%	38%	68%	68%	1%	1%	1%	0%	1%	0%	0%	0%
827	836	2	8	7.35-7.57	14%	25%	32%	44%	13%	26%	33%	48%	1%	0%	1%	1%	1%	0%	1%	2%
830	835	1	4	4.99-5.21	0%	1%	1%	14%	0%	1%	3%	23%	1%	1%	0%	0%	0%	0%	0%	1%
830	836	1	5	4.36-4.59	1%	1%	5%	25%	1%	1%	6%	32%	0%	0%	0%	1%	0%	0%	0%	1%
830	839	1	8	11.80-12.10	1%	8%	24%	38%	2%	9%	24%	41%	0%	0%	1%	0%	0%	0%	0%	1%
840	849	1	8	4.54-4.77	77%	77%	77%	76%	76%	78%	78%	74%	0%	2%	2%	1%	1%	1%	2%	1%
850	856	2	5	10.63-10.85	26%	49%	53%	64%	29%	51%	61%	65%	2%	1%	1%	2%	2%	0%	5%	1%
850	868	3	16	13.82-14.12	23%	41%	51%	64%	23%	42%	54%	64%	0%	0%	1%	1%	1%	0%	1%	1%
856	868	3	10	11.15-11.49	22%	37%	42%	42%	20%	37%	42%	40%	0%	1%	3%	0%	1%	0%	1%	2%
869	874	1	4	5.12-5.44	1%	0%	1%	7%	1%	1%	2%	4%	0%	0%	0%	1%	0%	0%	0%	2%
875	881	1	5	11.36-11.45	0%	0%	0%	0%	0%	0%	0%	0%	0%	0%	0%	0%	0%	0%	0%	0%
882	886	1	3	7.44-7.74	0%	0%	0%	0%	0%	0%	0%	0%	0%	0%	0%	0%	0%	0%	0%	0%
887	900	2	12	11.49-11.71	1%	5%	7%	7%	2%	7%	7%	7%	0%	0%	1%	0%	0%	0%	0%	1%
899	907	2	7	9.42-10.33	0%	4%	14%	18%	0%	4%	14%	18%	0%	0%	1%	1%	0%	0%	1%	0%
901	907	1	5	6.62-6.92	1%	5%	16%	19%	1%	5%	17%	17%	0%	0%	2%	1%	0%	0%	1%	1%
908	919	2	10	16.28-16.58	15%	34%	38%	37%	14%	36%	39%	36%	1%	1%	2%	1%	0%	0%	1%	2%
909	919	2	9	14.99-15.29	16%	38%	43%	40%	16%	40%	43%	39%	0%	2%	3%	1%	2%	1%	1%	1%
920	926	2	5	7.01-7.22	70%	74%	72%	68%	70%	71%	72%	69%	0%	0%	4%	4%	1%	4%	0%	3%
920	934	2	12	11.71-11.88	22%	31%	39%	48%	21%	35%	47%	49%	1%	2%	3%	1%	1%	0%	1%	2%
920	935	2	13	10.93-11.24	23%	32%	40%	53%	23%	41%	53%	55%	1%	1%	3%	0%	1%	0%	1%	2%
927	935	2	6	11.49-11.71	10%	23%	40%	76%	11%	42%	68%	81%	1%	1%	1%	0%	0%	0%	2%	2%

936	958	4	21	10.42-10.55	13%	19%	26%	27%	13%	24%	30%	32%	1%	1%	1%	0%	1%	0%	1%	1%
938	958	3	19	8.09-8.39	10%	15%	21%	22%	11%	20%	24%	24%	0%	1%	2%	0%	0%	0%	1%	2%
959	963	1	3	4.68-4.90	3%	2%	3%	2%	2%	3%	3%	2%	0%	0%	0%	0%	0%	0%	1%	0%
962	973	4	10	8.74-9.04	3%	7%	11%	15%	2%	7%	12%	14%	0%	0%	1%	2%	1%	0%	0%	2%
964	974	2	9	12.01-12.31	3%	7%	12%	15%	2%	7%	12%	14%	0%	0%	1%	0%	1%	0%	0%	1%
968	973	2	4	5.75-5.96	10%	18%	29%	37%	6%	18%	29%	37%	0%	0%	2%	0%	0%	0%	1%	1%
978	989	2	9	14.68-14.73	8%	35%	59%	56%	6%	34%	59%	55%	0%	2%	3%	0%	0%	0%	2%	2%
982	989	1	5	10.72-11.02	12%	47%	80%	79%	9%	46%	80%	75%	1%	0%	3%	1%	0%	1%	3%	2%
982	991	1	7	10.67-11.06	24%	56%	78%	76%	21%	57%	79%	74%	1%	0%	4%	0%	2%	0%	1%	5%
990	1002	2	11	11.88-12.01	12%	33%	44%	56%	7%	31%	41%	54%	1%	1%	1%	0%	1%	1%	0%	1%
992	997	1	4	7.01-7.31	24%	68%	71%	69%	11%	63%	71%	68%	1%	2%	3%	0%	1%	0%	1%	1%
1001	1009	1	7	7.48-7.79	35%	46%	56%	63%	30%	45%	55%	57%	1%	0%	4%	0%	1%	0%	1%	4%
1001	1019	4	17	12.40-12.62	11%	16%	24%	34%	10%	16%	24%	36%	0%	1%	1%	0%	0%	0%	1%	1%
1001	1023	4	21	13.13-13.43	8%	12%	19%	28%	7%	14%	26%	41%	0%	1%	1%	1%	0%	0%	1%	2%
1023	1033	3	9	9.86-10.16	34%	60%	62%	59%	60%	66%	64%	64%	2%	0%	2%	2%	1%	1%	0%	1%
1024	1033	3	8	9.47-9.68	33%	60%	60%	58%	59%	64%	64%	61%	4%	1%	3%	0%	2%	0%	1%	2%
1034	1044	2	9	3.15-3.39	51%	54%	54%	51%	48%	55%	55%	49%	1%	1%	3%	0%	1%	0%	0%	2%
1035	1044	3	8	3.15-3.39	53%	57%	57%	54%	51%	59%	59%	53%	1%	1%	3%	0%	2%	0%	0%	4%
