# Supplementary material for: Dynamic steps in receptor tyrosine kinase mediated activation of class IA phosphoinositide 3-kinases (PI3K) captured by H/D exchange (HDX-MS)
Source: Adv Biol Regul. 2013 Jan;53(1):97–110. doi: 10.1016/j.jbior.2012.09.005 (PMC3613897; doi:10.1016/j.jbior.2012.09.005)
Supplement: Supplementary file 5 [file mmc4.pdf]

GLOBAL HDX LEVELS

p85 alpha peptide

					p110 alpha / p85 alpha				p110 alpha / p85 alpha + pY				p110 beta / p85 alpha				p110 beta / p85 alpha + pY				p110 delta / p85 alpha				p110 delta / p85 alpha + pY			
Start	End	CS	#D	RT	3	30	300	3000	3	30	300	3000	3	30	300	3000	3	30	300	3000	3	30	300	3000	3	30	300	3000
6	13	1	6	10.27-10.57	1%	1%	4%	9%	1%	1%	4%	9%	1%	1%	4%	9%	1%	1%	3%	9%	1%	1%	4%	9%	0%	1%	4%	8%
8	13	1	4	8.94-9.02	1%	1%	6%	14%	1%	1%	5%	14%	1%	1%	6%	14%	1%	1%	5%	14%	1%	2%	8%	14%	1%	2%	6%	12%
14	21	2	6	3.11-3.67	24%	30%	35%	37%	23%	33%	36%	37%	24%	33%	35%	36%	23%	33%	35%	36%	25%	33%	36%	35%	22%	34%	37%	31%
22	30	2	7	14.93-15.10	1%	2%	8%	9%	1%	2%	8%	9%	1%	2%	8%	8%	1%	2%	8%	9%	1%	2%	8%	8%	1%	1%	8%	9%
25	30	1	4	11.18-11.44	1%	1%	1%	1%	1%	1%	0%	1%	0%	0%	0%	1%	1%	1%	0%	1%	0%	1%	0%	1%	0%	1%	0%	1%
31	37	1	5	5.86-5.99	74%	76%	79%	77%	72%	80%	77%	78%	74%	78%	78%	77%	71%	79%	77%	77%	74%	80%	80%	75%	70%	78%	80%	75%
38	52	2	12	9.93-10.27	27%	47%	59%	58%	26%	50%	59%	59%	28%	51%	57%	56%	28%	51%	58%	57%	28%	51%	59%	55%	25%	52%	60%	55%
53	60	1	6	14.20-14.46	4%	13%	22%	22%	3%	12%	21%	22%	5%	15%	21%	20%	4%	14%	21%	21%	4%	14%	22%	20%	2%	13%	22%	19%
53	72	2	17	13.68-13.90	3%	11%	19%	25%	3%	11%	18%	24%	4%	13%	19%	24%	3%	12%	18%	23%	4%	12%	19%	23%	3%	12%	19%	22%
53	73	2	18	14.33-14.63	3%	10%	17%	22%	3%	10%	16%	22%	3%	11%	16%	21%	3%	11%	16%	21%	3%	11%	17%	20%	3%	11%	17%	19%
61	72	2	9	8.33-8.46	4%	12%	18%	21%	3%	11%	17%	20%	3%	13%	17%	20%	2%	12%	17%	20%	4%	13%	18%	20%	2%	12%	17%	18%
73	102	4	19	7.47-8.07	54%	57%	61%	60%	53%	59%	61%	61%	53%	59%	60%	59%	53%	60%	61%	59%	53%	59%	62%	59%	50%	60%	62%	57%
73	106	4	23	7.25-7.55	49%	52%	55%	54%	49%	53%	55%	54%	48%	54%	53%	53%	48%	53%	54%	53%	48%	54%	55%	52%	46%	55%	56%	51%
77	106	4	19	5.64-6.12	58%	58%	59%	59%	57%	60%	58%	58%	56%	59%	57%	56%	56%	60%	58%	56%	56%	59%	59%	55%	53%	60%	60%	53%
108	112	1	3	3.62-3.86	89%	89%	89%	88%	88%	90%	89%	89%	83%	81%	82%	83%	82%	82%	83%	82%	82%	82%	83%	81%	82%	82%	83%	80%
133	146	3	12	11.09-11.39	6%	14%	29%	32%	5%	14%	28%	31%	6%	15%	28%	32%	5%	15%	28%	32%	7%	16%	29%	31%	6%	15%	29%	31%
133	149	2	15	12.51-12.82	14%	24%	41%	45%	13%	23%	40%	44%	14%	25%	41%	44%	13%	24%	40%	43%	14%	25%	41%	44%	12%	24%	41%	43%
138	146	1	7	6.07-6.29	10%	22%	43%	47%	9%	22%	42%	47%	10%	24%	43%	46%	9%	23%	42%	46%	9%	23%	43%	44%	7%	22%	43%	42%
149	158	2	8	7.38-7.73	46%	45%	52%	52%	51%	56%	60%	59%	44%	51%	51%	49%	49%	55%	56%	55%	42%	48%	52%	48%	44%	56%	59%	49%
150	157	1	6	3.44-3.67	69%	68%	71%	71%	68%	72%	70%	72%	70%	70%	71%	70%	69%	72%	71%	69%	71%	73%	73%	69%	66%	72%	73%	67%
150	158	1	7	6.34-6.55	55%	53%	54%	54%	58%	61%	58%	57%	53%	59%	54%	51%	56%	61%	56%	54%	52%	57%	55%	50%	51%	61%	59%	48%
150	160	1	9	5.99-6.34	50%	56%	61%	61%	51%	63%	65%	63%	50%	62%	61%	59%	52%	65%	63%	62%	48%	60%	62%	59%	47%	63%	66%	56%
162	173	1	9	10.36-10.62	41%	44%	50%	60%	40%	45%	48%	56%	40%	45%	48%	59%	39%	45%	46%	56%	39%	45%	49%	58%	36%	45%	48%	50%
175	185	1	9	12.43-12.64	8%	19%	23%	25%	8%	19%	25%	26%	11%	20%	27%	29%	10%	22%	28%	29%	10%	21%	27%	27%	9%	21%	28%	28%
177	185	2	7	10.75-10.96	8%	12%	13%	15%	8%	13%	14%	16%	9%	13%	14%	17%	9%	13%	15%	17%	9%	13%	15%	16%	8%	13%	15%	17%
177	202	3	21	17.57-17.88	7%	10%	11%	16%	7%	10%	11%	15%	7%	11%	11%	16%	7%	10%	11%	15%	7%	11%	12%	15%	6%	11%	12%	14%
177	203	3	22	17.83-18.14	7%	10%	11%	16%	6%	10%	11%	16%	7%	10%	10%	16%	6%	10%	10%	15%	7%	10%	11%	15%	6%	10%	11%	14%
186	202	2	12	13.90-14.20	10%	13%	15%	21%	9%	14%	15%	20%	10%	14%	15%	22%	10%	14%	15%	20%	10%	14%	15%	21%	9%	14%	15%	19%
187	202	2	11	13.46-13.64	10%	15%	16%	23%	10%	15%	15%	21%	10%	15%	16%	23%	10%	15%	15%	22%	11%	15%	16%	22%	9%	15%	16%	20%
207	217	1	8	9.93-10.19	48%	71%	78%	74%	46%	73%	75%	74%	49%	76%	74%	72%	46%	76%	74%	73%	48%	75%	76%	71%	43%	74%	77%	68%
219	237	4	15	12.77-13.16	4%	4%	8%	25%	4%	4%	7%	23%	4%	5%	9%	26%	4%	4%	8%	25%	3%	4%	9%	25%	3%	5%	8%	22%
223	237	3	11	10.10-10.32	6%	6%	10%	25%	6%	7%	9%	23%	6%	7%	10%	25%	6%	7%	9%	23%	5%	7%	11%	24%	5%	7%	10%	21%
223	261	4	35	16.09-16.57	6%	8%	11%	16%	5%	8%	10%	15%	6%	8%	10%	16%	5%	8%	10%	15%	6%	8%	11%	15%	5%	8%	11%	14%
238	247	3	8	15.96-16.18	0%	0%	-1%	0%	0%	0%	1%	-1%	0%	0%	0%	0%	0%	0%	1%	1%	0%	0%	0%	-1%	0%	1%	0%	-1%
238	250	3	9	17.35-17.57	0%	-1%	0%	0%	0%	0%	0%	0%	0%	-1%	0%	0%	0%	0%	0%	0%	0%	0%	0%	0%	0%	0%	0%	0%
238	259	3	20	16.35-16.57	4%	7%	11%	15%	4%	7%	11%	14%	4%	8%	11%	14%	4%	7%	11%	14%	4%	8%	12%	13%	3%	8%	12%	13%
238	261	4	22	16.48-16.79	9%	13%	17%	23%	9%	13%	17%	22%	10%	13%	17%	22%	9%	13%	17%	22%	9%	13%	18%	21%	9%	14%	17%	21%
241	261	3	19	12.34-12.60	12%	16%	21%	27%	11%	15%	20%	26%	11%	16%	20%	27%	11%	16%	20%	26%	11%	16%	21%	26%	10%	17%	21%	25%
244	261	2	16	9.89-10.10	9%	13%	19%	26%	9%	14%	19%	26%	9%	14%	19%	26%	10%	14%	19%	25%	9%	14%	20%	25%	9%	15%	20%	24%
262	272	2	8	15.36-16.05	1%	1%	3%	11%	0%	1%	3%	10%	1%	1%	4%	11%	1%	1%	3%	11%	0%	1%	3%	10%	0%	0%	3%	10%
273	286	1	12	10.96-11.35	41%	43%	53%	53%	43%	47%	54%	56%	41%	47%	52%	52%	43%	48%	54%	54%	39%	45%	54%	51%	40%	49%	56%	53%
287	293	2	5	13.07-13.38	0%	0%	0%	0%	0%	0%	0%	0%	0%	0%	0%	0%	0%	0%	0%	0%	0%	0%	0%	0%	0%	0%	0%	0%
294	325	3	23	8.72-9.19	71%	73%	73%	73%	70%	76%	73%	73%	70%	74%	71%	70%	70%	76%	72%	71%	69%	75%	73%	69%	66%	77%	74%	68%
294	326	3	24	9.32-9.63	70%	71%	72%	71%	69%	74%	72%	72%	69%	74%	70%	69%	69%	75%	71%	70%	68%	74%	72%	68%	65%	76%	73%	66%
294	328	3	26	10.23-10.79	69%	71%	71%	70%	69%	73%	71%	70%	68%	74%	69%	68%	69%	74%	70%	68%	67%	73%	72%	67%	64%	75%	72%	65%
329	341	2	11	14.20-14.50	17%	29%	43%	49%	17%	23%	37%	48%	20%	35%	43%	49%	18%	25%	37%	48%	20%	36%	45%	47%	16%	25%	39%	47%
329	355	3	25	14.20-14.50	11%	22%	35%	47%	10%	18%	32%	46%	14%	26%	38%	51%	11%	20%	33%	47%	16%	30%	44%	52%	11%	20%	36%	47%
333	341	2	7	14.24-14.67	20%	34%	50%	53%	19%	26%	43%	52%	23%	43%	51%	51%	20%	28%	43%	51%	24%	43%	53%	51%	18%	28%	45%	48%
334	341	2	6	11.01-11.09	23%	34%	53%	57%	23%	29%	45%	56%	27%	44%	58%	56%	24%	31%	45%	56%	28%	45%	58%	56%	22%	31%	48%	52%
334	355	2	20	12.82-12.99	12%	24%	37%	50%	11%	21%	34%	49%	16%	29%	41%	55%	13%	23%	36%	51%	19%	34%	50%	57%	13%	24%	38%	51%
342	349	2	6	5.14-5.28	5%	15%	28%	51%	4%	13%	27%	50%	6%	18%	31%	54%	4%	15%	30%	50%	10%	23%	40%	60%	4%	16%	33%	48%
346	355	2	8	7.73-7.86	9%	16%	19%	29%	8%	17%	19%	29%	14%	16%	19%	30%	10%	17%	19%	29%	16%	17%	24%	29%	10%	17%	21%	28%
356	370	2	13	8.68-8.81	16%	20%	25%	26%	16%	19%	24%	27%	17%	24%	25%	26%	15%	20%	25%	26%	18%	26%	26%	26%	15%	20%	26%	25%
356	371	2	14	7.86-8.16	17%	20%	25%	26%	16%	19%	23%	26%	18%	23%	26%	27%	15%	20%	24%	26%	18%	26%	26%	25%	15%	20%	26%	25%
356	372	3	15	10.70-11.01	13%	17%	20%	21%	14%	15%	20%	21%	15%	19%	22%	22%	14%	16%	21%	21%	16%	22%	24%	22%	14%	18%	23%	22%
371	380	2	8	4.08-4.30	6%	11%	17%	31%	9%	13%	16%	25%	11%	14%	20%	31%	10%	13%	17%	23%	10%	14%	23%	30%	9%	14%	19%	19%
372	380	2	7	3.67-3.86	6%	12%	20%	34%	10%	14%	19%	29%	12%	16%	23%	35%	12%	15%	20%	26%	11%	15%	25%	33%	10%	16%	20%	20%
373	380	1	6	3.39-3.57	7%	13%	19%	32%	12%	16%	18%	26%	13%	17%	23%	33%	13%	16%	18%	23%	13%	16%	24%	31%	12%	17%	20%	17%
381	398	3	15	12.56-12.77	17%	22%	26%	28%	11%	20%	24%	27%	17%	23%	25%	26%	10%	19%	24%	26%	15%	22%	26%	26%	8%	18%	24%	24%
381	400	3	17	12.17-12.25	16%	20%	27%	28%	11%	20%	26%	28%	17%	24%	26%	27%	11%	20%	25%	27%	15%	22%	28%	27%	8%	19%	26%	25%
401	413	2	11	10.53-10.79	7%	23%	25%	27%	5%	15%	24%	26%	7%	23%	25%	26%	5%	14%	22%	25%	15%	25%	25%	26%	5%	17%	25%	24%
401	413	3	11	10.53-10.75	7%	23%	25%	27%	5%	15%	25%	26%	7%	24%	25%	26%	5%	15%	22%	25%	15%	25%	25%	26%	5%	17%	25%	24%
402	413	3	10	9.76-9.97	8%	24%	26%	26%	5%	16%	24%	25%	8%	24%	25%	25%	5%	14%	21%	23%	16%	26%	26%	25%	5%	17%	25%	23%
403	413	2	9	10.57-10.70	8%	26%	29%	31%	5%	19%	28%	29%	9%	27%	29%	30%	5%	17%	25%	28%	18%	28%	29%	30%	5%	19%	29%	27%
414	420	2	4	8.12-8.42	40%	76%	84%	84%	9%	46%	80%	83%	42%	79%	84%	83%	6%	37%	71%	80%	68%	85%	84%	84%	11%	52%	81%	79%
421	434	2	11	10.66-10.88	22%	33%	42%	46%	22%	34%	41%	43%	25%	38%	42%	46%	23%	36%	41%	43%	26%	38%	42%	45%	23%	36%	41%	40%
444	456	2	11	7.86-8.07	0%	2%	4%	19%	1%	3%	11%	29%	1%	2%	10%	24%	1%	6%	25%	39%	1%	2%	10%	23%	1%	8%	26%	36%
444	466	4	22	8.76-9.07	0%	1%	2%	13%	0%	1%	5%	23%	0%	1%	6%	21%	0%	3%	18%	44%	0%	1%	6%	20%	0%	3%	17%	37%
457	466	2	8	6.60-6.73	0%	1%	1%	4%	0%	1%	2%	5%	1%	1%	3%	8%	1%	1%	5%	17%	0%	0%	3%	8%	0%	0%	3%	8%
467	476	1	8	6.34-6.69	1%	1%	8%	27%	1%	2%	10%	30%	1%	3%	19%	41%	1%	3%	19%	43%	1%	1%	7%	28%	1%	1%	8%	29%
470	476	1	5	3.76-3.90	1%	2%	12%	36%	1%	2%	14%	40%	1%	3%	23%	52%	1%	4%	25%	55%	1%	1%	9%	36%	1%	2%	11%	38%
477	486	1	8	7.12-7.29	0%	0%	2%	6%	0%	0%	2%	7%	1%	0%	2%	10%	1%	0%	3%	12%	2%	2%	4%	11%	1%	2%	3%	10%
480	486	1	4	4.61-4.70	0%	0%	0%	5%	0%	0%	0%	6%	0%	0%	1%	8%	0%	0%	1%	9%	0%	0%	1%	9%	0%	0%	1%	9%
487	494	2	6	13.46-13.64	-1%	-1%	0%	0%	-1%	-1%	-1%	-1%	-1%	-1%	-1%	-1%	-1%	-1%	0%	-1%	-1%	-1%	-1%	0%	-2%	-2%	-1%	-1%
508	537	4	28	10.40-10.66	3%	13%	21%	42%	3%	12%	22%	39%	4%	13%	22%	33%	4%	12%	21%	33%	5%	15%	28%	37%	4%	16%	27%	36%
508	537	5	28	10.40-10.66	3%	13%	21%	41%	3%	12%	21%	38%	4%	12%	21%	33%	4%	12%	21%	33%	6%	16%	28%	37%	5%	17%	27%	36%
532	537	1	4	3.72-3.86	0%	1%	6%	32%	0%	0%	6%	33%	0%	0%	4%	26%	0%	0%	4%	25%	0%	0%	10%	32%	0%	0%	10%	30%
538	555	3	15	8.38-8.59	0%	1%	5%	31%	0%	1%	5%	29%	0%	1%	9%	35%	0%	1%	13%	40%	0%	1%	12%	41%	0%	1%	11%	40%
538	556	3	17	10.01-10.27	0%	2%	6%	31%	1%	1%	7%	30%	1%	2%	10%	36%	1%	2%	14%	42%	1%	2%	13%	41%	1%	1%	11%	40%
556	570	2	12	8.76-9.11	0%	1%	3%	12%	0%	1%	4%	16%	0%	2%	8%	25%	0%	3%	13%	38%	0%	2%	5%	17%	0%	2%	7%	22%
571	581	3	9	8.03-8.33	1%	1%	2%	9%	1%	1%	3%	14%	1%	1%	5%	15%	1%	1%	10%	33%	0%	1%	5%	15%	0%	1%	8%	27%
582	596	3	13	7.99-8.25	13%	36%	48%	49%	15%	41%	48%	53%	12%	34%	47%	48%	18%	44%	50%	55%	14%	37%	48%	47%	22%	48%	54%	54%
582	601	2	18	11.61-11.65	8%	29%	41%	41%	9%	32%	43%	43%	10%	31%	41%	42%	14%	39%	46%	48%	10%	31%	43%	38%	15%	42%	46%	43%
610	637	4	25	8.38-8.76	14%	22%	31%	36%	8%	14%	16%	20%	13%	23%	26%	28%	8%	15%	16%	21%	13%	23%	27%	26%	7%	15%	17%	19%
610	638	3	26	9.58-9.97	13%	22%	31%	38%	8%	14%	15%	19%	13%	22%	26%	30%	8%	14%	15%	20%	12%	22%	28%	28%	7%	14%	16%	18%
614	637	4	21	7.86-8.03	15%	25%	36%	42%	8%	15%	18%	22%	15%	26%	30%	31%	8%	16%	17%	23%	14%	25%	31%	30%	7%	16%	18%	21%
637	646	2	8	6.42-6.64	7%	7%	16%	37%	7%	10%	11%	17%	7%	9%	12%	17%	7%	9%	10%	16%	6%	8%	12%	15%	6%	10%	11%	13%
638	646	1	7	4.83-5.10	9%	10%	18%	40%	9%	11%	12%	17%	9%	11%	15%	19%	9%	11%	13%	17%	9%	11%	14%	17%	8%	12%	14%	15%
638	647	2	8	6.42-6.51	7%	8%	16%	37%	7%	10%	11%	18%	7%	9%	12%	17%	7%	9%	11%	16%	7%	9%	13%	15%	6%	10%	12%	13%
647	656	2	8	3.86-4.08	43%	47%	54%	58%	26%	33%	41%	52%	44%	49%	54%	54%	27%	33%	44%	53%	45%	50%	56%	53%	25%	34%	44%	49%
647	657	3	9	5.68-5.90	32%	38%	46%	49%	19%	25%	31%	44%	31%	42%	45%	44%	19%	24%	32%	43%	31%	41%	46%	43%	18%	25%	32%	39%
648	656	2	7	3.35-3.44	54%	58%	68%	66%	32%	41%	49%	63%	55%	60%	65%	65%	33%	42%	52%	64%	54%	61%	66%	63%	31%	43%	52%	60%
648	657	2	8	4.74-4.79	37%	44%	52%	52%	21%	27%	34%	49%	36%	48%	53%	52%	22%	27%	36%	50%	37%	48%	53%	51%	19%	28%	35%	44%
661	680	2	18	8.38-8.59	15%	24%	35%	46%	10%	17%	23%	27%	15%	23%	28%	31%	10%	18%	23%	28%	15%	23%	28%	29%	9%	18%	24%	26%
661	687	3	24	12.00-12.34	17%	29%	39%	46%	12%	21%	30%	34%	12%	21%	28%	34%	11%	21%	29%	33%	12%	19%	28%	32%	11%	22%	31%	32%
661	691	3	28	13.90-14.93	20%	32%	42%	50%	16%	25%	32%	39%	16%	24%	30%	37%	15%	25%	32%	38%	15%	22%	30%	36%	14%	25%	33%	37%
662	680	3	17	7.68-7.94	18%	26%	38%	52%	11%	18%	25%	30%	18%	27%	31%	36%	11%	20%	26%	35%	17%	26%	30%	33%	10%	19%	26%	28%
662	687	3	23	11.82-12.08	18%	32%	42%	48%	13%	23%	32%	35%	13%	23%	29%	36%	13%	23%	31%	35%	13%	20%	29%	34%	12%	23%	32%	33%
681	687	1	4	12.60-12.86	41%	60%	69%	68%	29%	50%	66%	66%	20%	28%	42%	57%	27%	49%	64%	64%	18%	23%	41%	57%	26%	51%	67%	59%
688	693	1	4	5.95-6.03	29%	48%	54%	70%	27%	48%	49%	56%	32%	48%	50%	62%	29%	48%	49%	55%	33%	48%	51%	62%	27%	48%	49%	55%
692	696	1	3	9.97-10.14	1%	1%	2%	23%	1%	1%	1%	1%	1%	1%	1%	1%	1%	1%	1%	1%	1%	1%	1%	1%	1%	0%	1%	1%
692	703	2	10	9.02-9.11	3%	5%	13%	22%	2%	4%	4%	5%	1%	3%	4%	5%	2%	4%	4%	5%	1%	3%	4%	5%	2%	5%	5%	5%
694	703	3	8	9.58-9.80	4%	8%	17%	24%	3%	6%	7%	9%	2%	5%	6%	8%	3%	6%	7%	8%	2%	5%	6%	8%	3%	6%	8%	8%
697	703	2	5	5.68-5.99	8%	14%	30%	30%	4%	10%	11%	12%	2%	10%	13%	16%	4%	10%	11%	11%	3%	10%	12%	16%	4%	10%	11%	10%
704	710	2	5	5.77-6.12	16%	24%	37%	37%	1%	2%	10%	16%	16%	22%	37%	37%	1%	3%	13%	16%	16%	27%	36%	37%	1%	3%	13%	17%
711	719	1	6	12.90-13.25	2%	2%	15%	84%	2%	2%	7%	13%	2%	2%	8%	13%	2%	3%	9%	13%	2%	2%	8%	13%	2%	3%	9%	12%
711	724	2	11	9.80-10.06	29%	34%	51%	85%	27%	34%	41%	45%	28%	35%	41%	46%	27%	34%	41%	43%	29%	35%	41%	44%	26%	34%	43%	42%

STDEVS

		p110 alpha / p85 alpha				p110 alpha / p85 alpha + pY				p110 beta / p85 alpha				p110 beta / p85 alpha + pY				p110 delta / p85 alpha				p110 delta / p85 alpha + pY			
Start	End	3	30	300	3000	3	30	300	3000	3	30	300	3000	3	30	300	3000	3	30	300	3000	3	30	300	3000
6	13	0%	0%	0%	1%	0%	0%	0%	0%	0%	0%	0%	0%	0%	0%	0%	1%	0%	0%	1%	0%	0%	0%	0%	1%
8	13	0%	0%	0%	1%	0%	0%	0%	0%	0%	0%	0%	1%	0%	0%	0%	1%	0%	0%	1%	1%	0%	0%	0%	2%
14	21	0%	1%	1%	1%	0%	1%	0%	1%	0%	2%	1%	1%	1%	1%	1%	2%	1%	0%	3%	0%	0%	0%	0%	3%
22	30	0%	0%	1%	0%	0%	0%	0%	0%	0%	1%	0%	0%	0%	0%	0%	0%	0%	0%	1%	0%	0%	0%	0%	0%
25	30	1%	0%	0%	0%	0%	1%	0%	0%	0%	0%	0%	0%	1%	0%	0%	0%	0%	0%	0%	0%	1%	0%	0%	0%
31	37	1%	1%	3%	0%	1%	0%	0%	1%	1%	4%	1%	1%	1%	2%	0%	3%	1%	2%	3%	0%	1%	2%	1%	1%
38	52	1%	2%	1%	1%	1%	0%	1%	1%	0%	1%	1%	1%	1%	2%	0%	1%	2%	2%	4%	0%	1%	0%	1%	3%
53	60	1%	1%	0%	1%	0%	0%	0%	1%	0%	0%	0%	1%	0%	1%	0%	0%	0%	1%	2%	1%	0%	0%	1%	1%
53	72	1%	1%	0%	1%	0%	0%	0%	0%	0%	0%	0%	0%	0%	1%	0%	0%	0%	1%	1%	0%	0%	0%	1%	1%
53	73	0%	1%	0%	1%	0%	0%	0%	1%	0%	1%	0%	0%	0%	1%	0%	0%	0%	1%	2%	1%	0%	0%	1%	1%
61	72	0%	1%	0%	0%	0%	0%	0%	1%	0%	0%	0%	1%	2%	1%	0%	0%	0%	0%	1%	0%	2%	0%	0%	1%
73	102	1%	1%	0%	0%	0%	0%	1%	1%	0%	2%	1%	0%	1%	1%	0%	0%	0%	2%	3%	0%	1%	0%	2%	2%
73	106	0%	1%	0%	1%	0%	0%	1%	1%	0%	2%	0%	1%	2%	2%	0%	1%	0%	2%	3%	0%	1%	0%	2%	2%
77	106	0%	1%	1%	1%	0%	0%	0%	2%	1%	3%	0%	1%	2%	2%	0%	1%	1%	3%	3%	0%	1%	1%	1%	2%
108	112	1%	1%	0%	1%	1%	1%	0%	1%	0%	2%	1%	1%	1%	0%	1%	1%	1%	1%	1%	0%	0%	1%	1%	2%
133	146	0%	1%	0%	0%	0%	0%	1%	1%	0%	0%	1%	0%	0%	0%	0%	1%	0%	1%	1%	0%	0%	0%	0%	1%
133	149	0%	1%	0%	1%	0%	0%	0%	1%	0%	0%	0%	0%	0%	1%	0%	1%	0%	1%	0%	0%	0%	0%	1%	1%
138	146	0%	1%	1%	0%	0%	0%	0%	2%	0%	1%	0%	0%	0%	1%	0%	1%	1%	0%	3%	0%	0%	0%	1%	3%
149	158	0%	1%	1%	2%	1%	0%	1%	2%	2%	1%	1%	2%	3%	3%	0%	2%	1%	1%	6%	1%	2%	0%	2%	6%
150	157	1%	1%	1%	0%	1%	0%	1%	2%	1%	3%	1%	0%	1%	1%	2%	1%	0%	3%	2%	0%	0%	2%	2%	4%
150	158	0%	2%	2%	2%	1%	0%	2%	2%	2%	2%	2%	2%	4%	3%	0%	3%	2%	0%	6%	1%	1%	1%	1%	4%
150	160	0%	2%	2%	1%	1%	1%	2%	1%	1%	2%	1%	2%	2%	2%	0%	2%	2%	0%	5%	1%	1%	1%	1%	4%
162	173	0%	1%	1%	3%	0%	0%	1%	1%	1%	1%	0%	0%	2%	1%	0%	1%	0%	1%	3%	1%	1%	0%	1%	3%
175	185	1%	1%	2%	2%	1%	1%	1%	0%	0%	2%	1%	1%	0%	1%	0%	0%	0%	1%	0%	1%	0%	0%	1%	1%
177	185	0%	1%	0%	0%	0%	0%	0%	0%	0%	1%	0%	0%	0%	0%	0%	0%	0%	0%	0%	1%	0%	0%	0%	1%
177	202	0%	0%	0%	0%	0%	0%	0%	0%	0%	0%	0%	0%	0%	0%	0%	0%	0%	1%	1%	0%	0%	0%	0%	0%
177	203	0%	0%	0%	0%	0%	0%	0%	1%	0%	1%	0%	0%	0%	1%	0%	0%	0%	0%	0%	0%	0%	0%	1%	1%
186	202	0%	0%	0%	0%	0%	0%	0%	1%	0%	0%	0%	0%	0%	0%	0%	0%	0%	1%	1%	0%	0%	0%	1%	1%
187	202	0%	0%	0%	0%	0%	0%	0%	1%	0%	0%	0%	0%	0%	0%	0%	0%	0%	0%	1%	0%	1%	0%	0%	0%
207	217	2%	2%	2%	2%	0%	1%	1%	1%	1%	4%	0%	1%	3%	2%	1%	2%	2%	3%	5%	1%	2%	1%	2%	4%
219	237	0%	0%	0%	0%	0%	0%	0%	1%	0%	1%	0%	0%	0%	1%	0%	0%	0%	0%	1%	0%	0%	0%	0%	1%
223	237	0%	0%	0%	1%	0%	0%	0%	1%	0%	1%	0%	0%	1%	1%	0%	0%	0%	1%	1%	0%	0%	0%	0%	1%
223	261	0%	0%	0%	0%	0%	0%	0%	1%	0%	0%	0%	0%	0%	1%	0%	0%	0%	0%	0%	0%	0%	0%	0%	0%
238	247	1%	0%	1%	0%	1%	1%	0%	0%	0%	1%	1%	0%	0%	1%	1%	0%	1%	0%	0%	0%	0%	1%	1%	1%
238	250	0%	0%	1%	0%	0%	1%	0%	0%	0%	0%	0%	1%	0%	0%	0%	0%	0%	0%	1%	0%	0%	0%	1%	0%
238	259	0%	0%	0%	0%	0%	0%	0%	0%	0%	1%	0%	0%	0%	1%	0%	0%	0%	1%	1%	0%	0%	0%	1%	1%
238	261	0%	0%	0%	0%	0%	0%	0%	1%	0%	1%	0%	0%	0%	1%	0%	0%	0%	1%	1%	0%	0%	0%	1%	1%
241	261	0%	0%	0%	1%	0%	0%	1%	1%	0%	1%	0%	0%	0%	1%	0%	0%	0%	1%	1%	0%	0%	0%	0%	1%
244	261	0%	1%	0%	0%	1%	0%	0%	1%	1%	1%	0%	0%	0%	1%	0%	0%	0%	0%	1%	0%	0%	1%	2%	0%
262	272	0%	0%	0%	0%	0%	0%	0%	0%	0%	0%	0%	0%	0%	0%	0%	0%	0%	0%	0%	0%	0%	0%	0%	0%
273	286	0%	1%	0%	1%	0%	1%	1%	1%	1%	3%	0%	0%	2%	2%	0%	0%	1%	3%	3%	0%	1%	0%	1%	2%
287	293	0%	0%	0%	0%	0%	0%	0%	0%	0%	0%	0%	0%	0%	1%	0%	0%	0%	0%	0%	0%	0%	0%	0%	0%
294	325	0%	2%	0%	2%	1%	0%	1%	2%	2%	2%	1%	1%	2%	1%	0%	1%	1%	2%	3%	1%	1%	0%	1%	2%
294	326	0%	2%	0%	2%	0%	1%	1%	2%	2%	2%	0%	1%	2%	2%	0%	0%	1%	3%	4%	1%	2%	0%	1%	2%
294	328	1%	2%	1%	1%	1%	1%	1%	1%	2%	2%	1%	0%	2%	2%	0%	1%	0%	2%	4%	1%	1%	0%	1%	2%
329	341	0%	2%	1%	1%	0%	0%	1%	2%	0%	1%	1%	0%	1%	1%	0%	0%	1%	2%	2%	0%	1%	0%	1%	1%
329	355	0%	1%	1%	0%	0%	0%	0%	1%	0%	1%	1%	0%	0%	1%	0%	0%	1%	1%	1%	1%	0%	1%	1%	1%
333	341	1%	2%	0%	1%	0%	0%	0%	1%	1%	1%	0%	0%	1%	2%	0%	1%	0%	1%	2%	0%	0%	1%	1%	2%
334	341	0%	2%	2%	1%	0%	0%	1%	1%	0%	2%	2%	1%	1%	2%	0%	1%	1%	1%	4%	1%	1%	1%	2%	3%
334	355	1%	1%	0%	0%	0%	0%	1%	1%	0%	1%	0%	1%	1%	1%	0%	0%	0%	1%	2%	0%	0%	0%	1%	1%
342	349	1%	0%	0%	1%	0%	1%	0%	1%	0%	1%	1%	1%	0%	1%	0%	1%	1%	0%	3%	0%	0%	0%	1%	2%
346	355	0%	0%	0%	0%	0%	0%	0%	1%	0%	1%	0%	0%	0%	1%	0%	1%	0%	1%	2%	0%	1%	0%	0%	1%
356	370	0%	1%	1%	0%	0%	0%	0%	1%	1%	2%	0%	0%	1%	1%	0%	1%	1%	1%	1%	1%	1%	1%	0%	1%
356	371	0%	0%	0%	0%	0%	0%	1%	0%	0%	1%	0%	0%	0%	1%	1%	1%	1%	1%	2%	1%	1%	0%	0%	1%
356	372	1%	1%	1%	1%	1%	0%	0%	0%	0%	2%	0%	0%	0%	1%	0%	0%	1%	1%	1%	1%	0%	0%	1%	1%
371	380	0%	1%	0%	0%	0%	2%	0%	1%	1%	1%	0%	1%	1%	1%	0%	1%	1%	1%	3%	1%	0%	0%	0%	2%
372	380	1%	2%	1%	0%	0%	2%	0%	1%	0%	0%	1%	0%	1%	1%	0%	1%	0%	0%	2%	1%	0%	0%	0%	3%
373	380	0%	0%	1%	0%	0%	1%	0%	1%	0%	2%	1%	1%	1%	1%	1%	2%	1%	1%	2%	0%	0%	0%	0%	4%
381	398	0%	1%	0%	2%	0%	0%	0%	1%	0%	1%	0%	0%	1%	1%	0%	0%	0%	1%	1%	0%	0%	0%	1%	1%
381	400	0%	1%	0%	1%	0%	1%	1%	1%	0%	2%	0%	0%	1%	1%	0%	0%	0%	1%	2%	0%	0%	0%	1%	1%
401	413	0%	1%	0%	1%	0%	0%	0%	1%	0%	1%	1%	0%	0%	1%	0%	0%	1%	1%	1%	0%	0%	0%	1%	1%
401	413	1%	1%	0%	1%	0%	0%	0%	0%	0%	1%	0%	0%	0%	1%	0%	0%	1%	1%	1%	0%	0%	0%	0%	1%
402	413	0%	1%	0%	1%	0%	0%	0%	1%	0%	1%	0%	0%	0%	1%	0%	0%	1%	1%	1%	0%	0%	0%	0%	1%
403	413	0%	1%	0%	1%	0%	1%	0%	1%	1%	1%	0%	0%	0%	1%	0%	0%	2%	2%	1%	0%	0%	0%	1%	2%
414	420	1%	2%	0%	1%	0%	0%	1%	2%	0%	1%	1%	1%	0%	0%	0%	1%	2%	0%	1%	3%	1%	1%	0%	2%
421	434	1%	1%	1%	3%	1%	0%	1%	1%	1%	1%	0%	1%	0%	1%	0%	0%	2%	2%	2%	0%	0%	1%	0%	2%
444	456	0%	2%	2%	4%	0%	0%	0%	0%	0%	0%	1%	2%	0%	0%	1%	0%	0%	1%	0%	1%	0%	0%	1%	3%
444	466	0%	0%	0%	1%	0%	0%	0%	1%	0%	0%	1%	1%	0%	0%	0%	1%	0%	0%	0%	0%	0%	0%	0%	1%
457	466	0%	0%	0%	0%	0%	0%	0%	0%	0%	0%	0%	1%	0%	0%	0%	1%	0%	0%	1%	0%	0%	0%	0%	1%
467	476	0%	2%	2%	2%	0%	1%	0%	0%	0%	0%	1%	2%	0%	0%	0%	2%	0%	0%	1%	0%	1%	0%	1%	1%
470	476	0%	1%	3%	4%	0%	0%	0%	0%	0%	0%	1%	1%	0%	0%	1%	0%	0%	0%	2%	1%	0%	0%	1%	1%
477	486	1%	1%	0%	1%	0%	0%	1%	0%	0%	0%	0%	1%	0%	0%	0%	1%	0%	1%	0%	0%	0%	0%	1%	1%
480	486	0%	0%	0%	1%	0%	0%	0%	1%	0%	0%	0%	0%	0%	0%	0%	0%	0%	0%	0%	0%	0%	0%	0%	1%
487	494	0%	0%	0%	0%	0%	0%	1%	0%	1%	1%	1%	0%	0%	1%	0%	0%	0%	0%	0%	0%	0%	0%	0%	1%
508	537	0%	1%	2%	1%	0%	0%	1%	0%	0%	1%	0%	0%	0%	1%	0%	0%	1%	2%	1%	0%	0%	0%	0%	1%
508	537	0%	1%	2%	2%	0%	0%	0%	0%	0%	1%	0%	0%	1%	1%	0%	0%	0%	1%	1%	0%	0%	0%	1%	2%
532	537	0%	1%	1%	3%	0%	0%	0%	0%	0%	1%	0%	1%	0%	0%	0%	1%	0%	0%	0%	1%	0%	0%	0%	1%
538	555	0%	1%	1%	1%	0%	0%	0%	0%	0%	0%	1%	2%	0%	0%	0%	0%	0%	0%	0%	0%	0%	0%	0%	0%
538	556	0%	0%	1%	0%	0%	1%	0%	1%	1%	0%	1%	1%	0%	0%	0%	1%	0%	0%	1%	1%	0%	0%	0%	1%
556	570	0%	1%	1%	2%	0%	0%	0%	0%	0%	0%	1%	3%	0%	0%	0%	1%	0%	0%	1%	0%	0%	0%	0%	1%
571	581	0%	0%	0%	0%	0%	0%	0%	0%	0%	0%	1%	1%	0%	0%	1%	1%	0%	0%	0%	0%	0%	0%	0%	3%
582	596	1%	0%	1%	1%	0%	0%	1%	1%	0%	2%	0%	1%	1%	3%	1%	1%	1%	2%	3%	0%	0%	0%	1%	3%
582	601	2%	5%	2%	4%	0%	2%	1%	0%	0%	3%	1%	2%	1%	1%	0%	1%	0%	4%	4%	1%	0%	0%	2%	3%
610	637	0%	1%	0%	1%	0%	0%	0%	0%	0%	1%	0%	0%	1%	1%	0%	1%	1%	2%	2%	0%	0%	0%	1%	1%
610	638	1%	1%	0%	0%	0%	0%	1%	0%	0%	1%	1%	1%	0%	2%	0%	0%	0%	1%	1%	0%	1%	0%	1%	1%
614	637	0%	1%	0%	1%	0%	0%	0%	1%	0%	2%	0%	1%	0%	1%	0%	0%	1%	2%	2%	0%	0%	0%	1%	1%
637	646	0%	0%	1%	2%	0%	0%	0%	1%	0%	1%	0%	1%	1%	2%	0%	1%	0%	0%	2%	1%	0%	0%	0%	2%
638	646	0%	0%	0%	1%	0%	1%	0%	1%	0%	1%	0%	1%	0%	1%	0%	1%	0%	1%	2%	0%	0%	0%	1%	2%
638	647	0%	0%	1%	1%	0%	0%	0%	1%	0%	1%	0%	1%	1%	2%	0%	1%	0%	0%	2%	1%	0%	0%	0%	2%
647	656	1%	1%	0%	0%	0%	1%	0%	1%	0%	2%	1%	1%	1%	1%	0%	1%	0%	2%	3%	0%	1%	0%	0%	3%
647	657	0%	1%	1%	2%	0%	1%	1%	2%	1%	1%	1%	2%	1%	2%	0%	2%	2%	1%	4%	0%	0%	0%	1%	2%
648	656	1%	0%	2%	0%	0%	0%	1%	1%	0%	3%	1%	1%	1%	1%	1%	0%	1%	3%	2%	0%	1%	0%	1%	3%
648	657	0%	1%	1%	0%	0%	1%	0%	2%	1%	2%	1%	1%	1%	2%	0%	1%	0%	2%	3%	0%	0%	0%	0%	3%
661	680	0%	1%	0%	1%	0%	0%	0%	1%	0%	1%	0%	0%	0%	1%	0%	1%	1%	1%	2%	1%	0%	0%	1%	2%
661	687	0%	1%	0%	1%	0%	0%	0%	1%	0%	1%	0%	0%	0%	1%	0%	0%	0%	1%	2%	0%	0%	0%	1%	2%
661	691	0%	2%	0%	1%	0%	0%	0%	1%	0%	1%	0%	0%	0%	1%	0%	0%	0%	1%	2%	0%	0%	0%	0%	1%
662	680	1%	1%	0%	1%	0%	0%	0%	1%	0%	1%	0%	1%	0%	1%	0%	4%	1%	1%	2%	0%	0%	0%	1%	3%
662	687	0%	1%	0%	1%	0%	0%	0%	1%	0%	2%	0%	0%	0%	1%	0%	0%	0%	2%	2%	1%	0%	0%	1%	1%
681	687	1%	3%	0%	2%	0%	0%	0%	2%	0%	1%	0%	0%	1%	2%	1%	1%	0%	1%	3%	3%	1%	0%	1%	4%
688	693	1%	0%	0%	1%	0%	0%	0%	1%	1%	0%	0%	1%	1%	1%	0%	0%	2%	1%	0%	1%	1%	0%	0%	3%
692	696	0%	0%	0%	1%	0%	1%	0%	0%	0%	0%	0%	0%	0%	0%	0%	0%	0%	0%	0%	0%	0%	0%	0%	0%
692	703	0%	0%	0%	1%	0%	0%	0%	0%	0%	0%	0%	0%	0%	0%	0%	0%	0%	0%	1%	0%	0%	0%	0%	0%
694	703	0%	0%	0%	1%	0%	0%	0%	0%	0%	0%	0%	1%	0%	1%	1%	0%	0%	0%	1%	0%	0%	0%	0%	1%
697	703	0%	0%	6%	2%	0%	1%	0%	0%	0%	1%	0%	1%	0%	0%	0%	1%	0%	0%	1%	0%	0%	1%	1%	1%
704	710	0%	1%	1%	0%	0%	0%	0%	1%	1%	1%	0%	0%	0%	0%	0%	0%	1%	1%	3%	0%	0%	0%	0%	0%
711	719	0%	0%	2%	1%	0%	0%	0%	0%	0%	0%	0%	0%	0%	0%	0%	0%	0%	0%	1%	0%	0%	0%	0%	0%
711	724	0%	1%	0%	0%	0%	0%	0%	0%	0%	1%	0%	1%	1%	1%	0%	0%	0%	1%	2%	0%	1%	0%	1%	1%

0.68% average % error
